# LaeA Control of Velvet Family Regulatory Proteins for Light-Dependent Development and Fungal Cell-Type Specificity

**DOI:** 10.1371/journal.pgen.1001226

**Published:** 2010-12-02

**Authors:** Özlem Sarikaya Bayram, Özgür Bayram, Oliver Valerius, Hee Soo Park, Stefan Irniger, Jennifer Gerke, Min Ni, Kap-Hoon Han, Jae-Hyuk Yu, Gerhard H. Braus

**Affiliations:** 1 Institute of Microbiology and Genetics, Department of Molecular Microbiology and Genetics, Georg August University, Göttingen, Germany; 2 Departments of Bacteriology and Genetics, University of Wisconsin-Madison, Madison, Wisconsin, United States of America; 3 Department of Pharmaceutical Engineering, Woosuk University, Wanju, Korea; Leibniz Institute for Natural Product Research and Infection Biology, Germany

## Abstract

VeA is the founding member of the velvet superfamily of fungal regulatory proteins. This protein is involved in light response and coordinates sexual reproduction and secondary metabolism in *Aspergillus nidulans*. In the dark, VeA bridges VelB and LaeA to form the VelB-VeA-LaeA (velvet) complex. The VeA-like protein VelB is another developmental regulator, and LaeA has been known as global regulator of secondary metabolism. In this study, we show that VelB forms a second light-regulated developmental complex together with VosA, another member of the velvet family, which represses asexual development. LaeA plays a key role, not only in secondary metabolism, but also in directing formation of the VelB-VosA and VelB-VeA-LaeA complexes. LaeA controls VeA modification and protein levels and possesses additional developmental functions. The *laeA* null mutant results in constitutive sexual differentiation, indicating that LaeA plays a pivotal role in inhibiting sexual development in response to light. Moreover, the absence of LaeA results in the formation of significantly smaller fruiting bodies. This is due to the lack of a specific globose cell type (Hülle cells), which nurse the young fruiting body during development. This suggests that LaeA controls Hülle cells. In summary, LaeA plays a dynamic role in fungal morphological and chemical development, and it controls expression, interactions, and modification of the velvet regulators.

## Author Summary

Numerous fungi have the potential to infect immunocompromised patients or to contaminate and spoil our nutrients. They represent an increasing danger that threatens public health and agriculture. This requires improved understanding of fungal growth, development, dissemination of spores, and mycotoxin production. We have discovered two related fungal specific protein complexes that provide a molecular link among spore formation, fungal development, and secondary metabolite production. The subunit allocation of both complexes depends on each other, and they share a common subunit. These complexes comprise three related and in fungi conserved proteins of the velvet family that function in concert with a known regulator of secondary metabolism, LaeA. This protein controls the formation of both complexes but is only a part of the trimeric complex. We found that this regulator of secondary metabolism also possesses several developmental control functions in gene expression. These protein complexes discovered in the fungal model system *Aspergillus nidulans* are conserved in fungal pathogens where they might provide novel insights for understanding growth, development, and interaction with their respective hosts.

## Introduction

Multicellular organisms have developed a variety of different cell types, which become apparent during the ontogenesis of an organism to its adult form. Cell differentiation requires the coordinated interplay of key regulators, which respond to internal and external cues. Cell type specificity often requires specific physiology and metabolism to allow the formation of tissues and organs exhibiting various functions for the organism. Early cells are often omnipotent or pluripotent and lose potential during differentiation except for those misregulated or uncontrolled for cell-differentiation, which might result in tumorogenesis or cancer [1].

Higher fungi produce a limited number of specialized cells and serve as simple and easily tractable models to study cell differentiation. Filamentous fungi grow by forming polar hyphae where similar cellular units are reiterated. The tip as well as branch points of the filamentous hyphae show increased cellular activity. Highly specialized cells include the ubiquitous asexual or sexual spores that are often dispersed into the air for propagation, and specialized cells that are required to form spores. Especially, sexual spore formation can require complicated fruiting bodies consisting of additional specialized cells that form various tissues [2]–[4]. Furthermore fungal differentiation is coupled to the production of various secondary metabolites including mycotoxins and antibiotics [5], which are assumed to provide a chemical shield against competitors [6].

**Figure 1:**
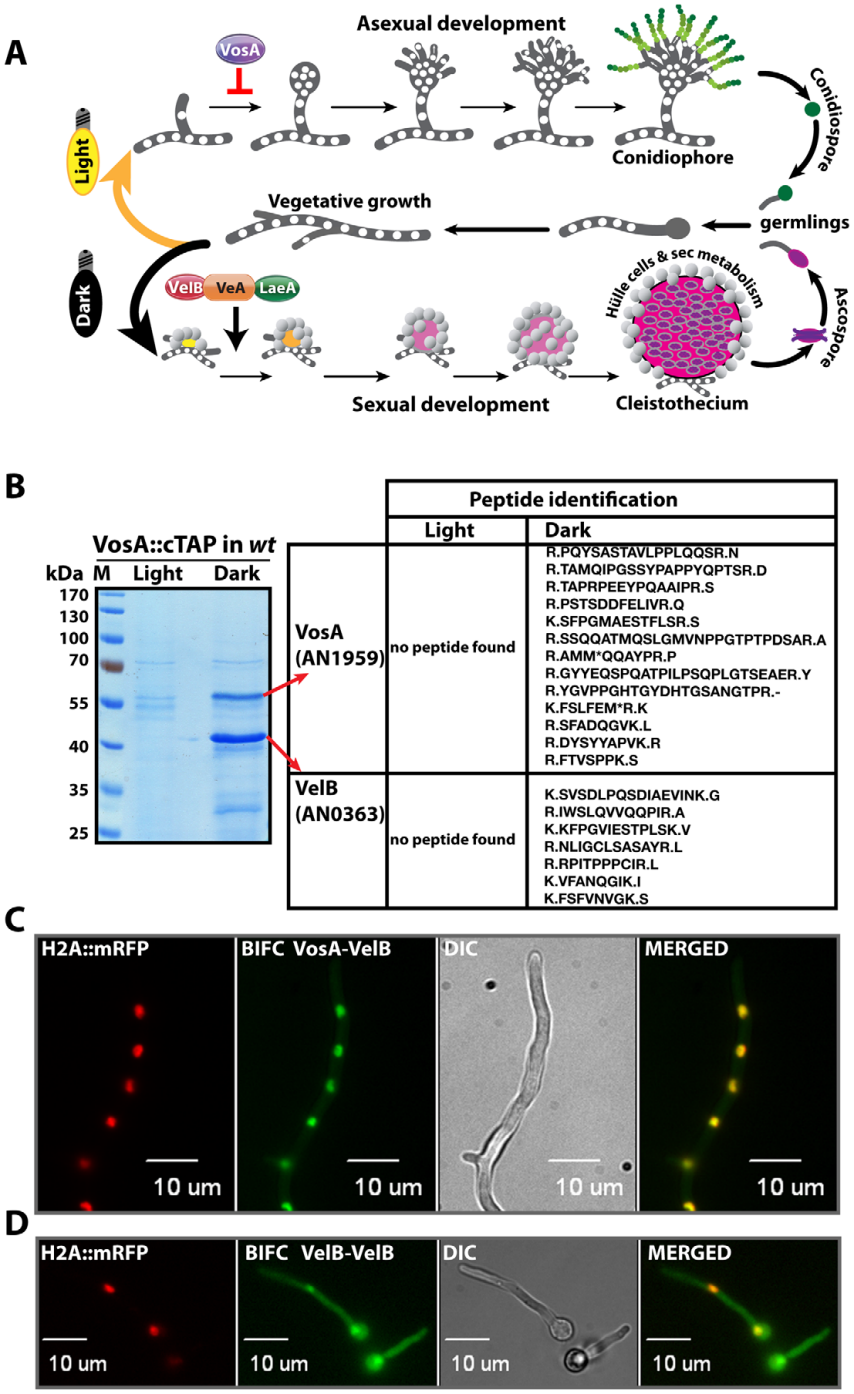
Life cycle of *Aspergillus nidulans* and identification of the VosA-associated proteins by tandem affinity purification. (A) *Aspergillus nidulans* can grow as a filament (vegetative growth). Light favors asexual development and results in asexual spores (conidiospores) produced by conidiophores. Asexual development is repressed by VosA protein. Darkness favors sexual development and requires the trimeric VelB-VeA-LaeA complex. This leads to fruiting bodies (cleistothecia) nursed by Hülle cells. Meiotically produced sexual spores (ascospores) are formed within the fruiting bodies. White round dots indicate the haploid nuclei of the fungus. (B) SDS-polyacrylamide (10%) gel electrophoresis of TAP enrichment for VosA stained with brilliant blue G. Polypeptides identified from the bands of affinity purification from the light and dark grown cultures are shown (Table S4). (C) Bimolecular fluorescence complementation (BIFC) in vegetative hyphae with enriched nuclear interaction of the VosA-VelB heterodimer. The N-terminal half of the enhanced yellow fluorescent protein (EYFP) fused to the N-terminus of the VosA protein (N-EYFP::VosA) interacts with the C-terminal half of EYFP fused to VelB (C-EYFP::VelB) *in vivo*. Histone 2A monomeric red fluorescent protein fusion (H2A::mRFP) visualizes the nuclei. (D) BIFC of the VelB-VelB homodimer formation in the cytoplasm and nuclei. N-EYFP::VelB interacts with C-EYFP::VelB.

The model fungus *Aspergillus nidulans* grows vegetatively as a filament with two developmental options: it can either enter the asexual or the sexual developmental pathway (Figure 1A). Sexual development produces closed spherical fruiting bodies (cleistothecia) where meiotic sexual spores are generated. The maturing fruiting body is embedded in a tissue of globose Hülle cells that are proposed to provide protection and nourishment [7]. The molecular mechanism triggering the developmental switch from a vegetative to globose fungal cell is presently unknown [4], [8].

Formation of sexual fruiting bodies and production of certain secondary metabolites occur preferentially in darkness in *A. nidulans* and are coordinately inhibited by light as an external signal [9], [10]. In contrast, formation of the asexual spores is promoted by light. Light is perceived by various receptors [11] including the red light receptor FphA [12], the blue light receptors LreA-LreB [13] or the blue-UVA receptor CryA [14]. The molecular mechanism of light signal transduction is yet unknown as well as the exact function of the conserved VeA (*ve*lvet *A*) protein, which is the founding member of the velvet family [9]. CryA controls the levels of the VeA mRNA [14], whereas FphA, LreB and LreA act through physical interaction with VeA by a yet unknown molecular mechanism [12], [13]. Strains lacking *veA* fail to produce cleistothecia and undergo asexual sporulation under both light and dark conditions.

VeA is a part of the heterotrimeric velvet complex [9], which is assembled in the nucleus in darkness and contains the VeA-related developmental regulator VelB (*ve*lvet-*l*ike B) and LaeA, the global regulator of secondary metabolism [15]. All three proteins are conserved in various fungi [16]–[19]. VelB interacts with the N-terminus of VeA, whereas LaeA interacts with the C-terminus of VeA. Illumination reduces the cellular amounts of VeA [9]. VelB and LaeA are unable to interact with each other and need VeA as a bridging factor. In addition, VeA supports the transport of VelB into the nucleus, whereas nuclear localization of LaeA does not depend on the other subunits of the velvet complex. This suggests that the complex fulfills its function in coordinating sexual development and secondary metabolism in darkness primarily by controlling gene expression in the nucleus [9].

In this study, we show that the coordination of development and secondary metabolism is only one function of the velvet complex subunits. VelB is a part of a second novel light-regulated complex, which includes VosA (*v*iability *o*f *s*pores A). VeA, VelB and VosA are related members of the fungus-specific novel velvet family regulatory proteins [16]. The VelB-VosA complex can repress asexual development and is essential for asexual as well as sexual spore maturation and trehalose biogenesis. Moreover, besides being a global regulator of secondary metabolism, LaeA executes three important novel developmental functions: (i) LaeA controls the VelB complex allocation between VosA-VelB and VeA-VelB. (ii) LaeA is required for the transition from filamentous cells to globular Hülle cells, and (iii) LaeA is a key factor in light control of fungal development.

## Results

### Identification of an alternative light-regulated protein complex, VelB-VosA

Functionally tagged versions of all three proteins of the velvet complex VelB-VeA-LaeA are able to recruit the respective other subunits from a fungal protein extract. In addition, the phenotypes of the corresponding *velB* or *veA* deletion strains are similar: both mutants are unable to perform sexual development and are impaired in light control and secondary metabolism [9]. However, only a tagged VelB, but neither VeA nor LaeA, is able to recruit another related protein, VosA [9]. VosA was isolated as a high copy repressor of asexual development and is also required for spore maturation, trehalose biogenesis and long-term viability of asexual and sexual spores [16]. We analyzed whether VelB has an additional yet unexplored function in fungal development.

We initially examined whether VosA is the fourth subunit of the velvet complex during the establishment of developmental competence. Developmental competence describes the phenomenon that *A. nidulans* spores require at least 20 hours of growth after germination to respond to external signals when placed on the surface of a medium [20]. *A. nidulans* strain expressing a functional *vosA::ctap* fusion driven by its native promoter was cultivated in liquid medium and induced on the surface of solid medium for asexual or sexual development by incubation in light and dark, respectively. Purification of VosA::cTAP was performed from 12 hours post- induction cultures on surface of solid medium after developmental competence was achieved. Tagged VosA was only present in the dark and co-purified exclusively with the VelB protein, but neither with VeA nor LaeA (Figure 1B and Table S4). VelB is not only a part of the VelB-VeA-LaeA velvet complex, but also a part of the second complex VelB-VosA when developmental competence is established.

Heterologous expression of VelB in *Escherichia coli* resulted primarily in dimers suggesting that VelB is able to form homodimers (data not shown) in addition to the VelB-VosA heterodimer. We employed a split-YFP system to determine the *in vivo* compartment where the subunits of the VelB-VosA heterodimer or of the VelB-VelB homodimer interact. An mRFP histone fusion served as control to track the nuclei within the hyphae. The VosA-VelB YFP signal colocalized predominantly to the nuclear RFP signal, indicating that the VosA-VelB complex is formed in the nucleus (Figure 1C). In contrast, we found the combined signal of N-YFP::VelB and C-YFP::VelB *in vivo* in the cytoplasm as well as in the nucleus (Figure 1D).

These data suggest that VelB is not only a component of the nuclear VelB-VeA-LaeA complex, but can also (i) form a VelB homodimer in the cytoplasm as well as in the nucleus, and (ii) be part of the nuclear VosA-VelB heterocomplex, which is hardly detectable in the cytoplasm.

### The role of VelB in fungal spore maturation

**Figure 2:**
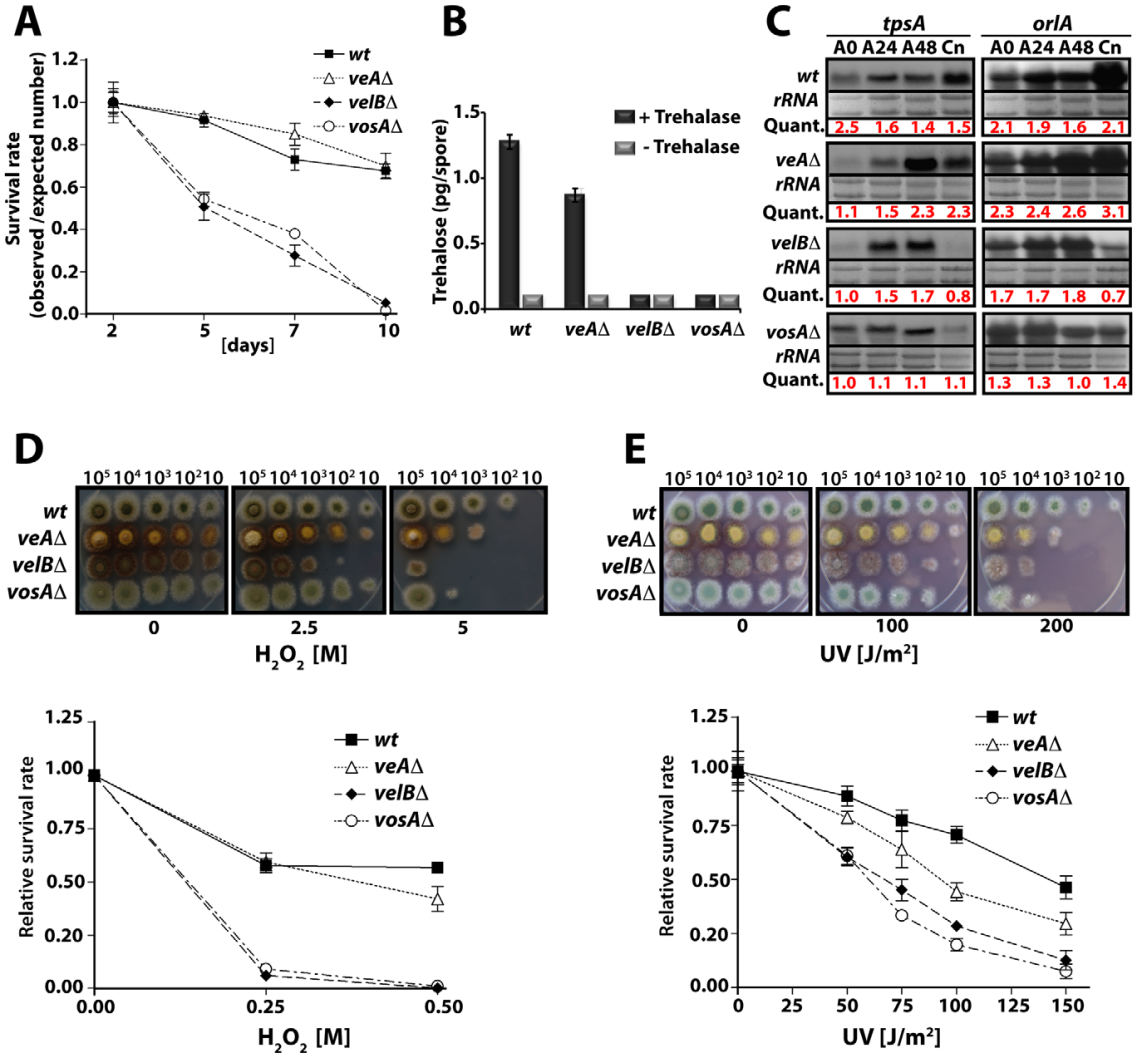
VelB function in spore viability and trehalose biogenesis. (A) Viability of wild type and velvet mutant strains conidia grown at 37°C for 2, 5, 7, and 10 days. (B) Amount of trehalose (pg) per conidium in the 2 day old conidia of wild type and the *velvet* deletion mutants (measured in triplicate). Samples without the trehalase treatment served as controls. (C) Levels of *tpsA* and *orlA* transcripts in wild type and *velvet* mutant strains. Numbers indicate the time (hour) of incubation in post-asexual (A) developmental induction and (Cn) represents conidia. Equal loading of total RNA was evaluated by ethidium bromide staining of rRNA. Quantification of *tpsA* and *orlA* expression levels are indicated at the bottom of the blots. Quant: Quantification. (D) Tolerance of the conidia of wild type and *velvet* mutant strains against H_2_O_2_ (see text). (E) Tolerance of the conidia of wild type and *velvet* mutant strains against ultra violet (UV) irradiation.

VosA is not only a high-copy repressor of asexual development but also plays an essential role in the maturation and viability of spores primarily by coupling trehalose biogenesis and sporogenesis [16]. We analysed whether VelB plays a similar role, as it forms the nuclear VelB-VosA heterodimeric complex. The viability of spores, trehalose biosynthesis and tolerance against various stresses were compared between the *velB*Δ, wild type, and *veA*Δ or *vosA*Δ strains (Figure 2A). The conidia of both *velB*Δ and *vosA*Δ strains displayed severe viability defects, whereas viability of the *veA*Δ conidia was similar to that of wild type, indicating that VelB and VosA play a specific role in conferring spore viability. VelB is needed for the proper biogenesis of trehalose in conidia, because trehalose was undetectable in the *velB*Δ and *vosA*Δ conidia (Figure 2B). The mRNA levels of two genes (*tpsA* and *orlA*) associated with trehalose synthesis [21], [22] revealed that the *velB*Δ and *vosA*Δ strains both exhibited reduced *tpsA* and *orlA* transcript levels during the late phase of development and in conidia (Figure 2C). These results indicate that both VelB and VosA are necessary for trehalose biogenesis and viability of spores.

As trehalose plays an important protective role in response to various stresses, we tested whether the absence of *velB* would result in decreased tolerance of the spores against various stresses, and examined two-day old conidia of wild type, *veA*Δ, *velB*Δ, and *vosA*Δ strains. Serially diluted spores were cultivated on solid medium containing various H_2_O_2_ concentrations. The *velB*Δ conidia were the most sensitive among those tested (Figure 2D). At 0.25 M H_2_O_2_, 90% of the *velB*Δ conidia were non-viable, whereas only about 40% of wild type and the *veA*Δ conidia lost viability. After being treated with 0.5 M H_2_O_2_, most of the *velB*Δ and *vosA*Δ conidia were non-viable, whereas about 60% and 50% of wild type and the *veA*Δ conidia, respectively, were viable (Figure 2D). These data were further confirmed by testing the tolerance against UV, where both the *vosA*Δ and *velB*Δ conidia were more sensitive than those of wild type. Being exposed to 100 J/m^2^ UV only about 30% of the *velB*Δ and *vosA*Δ conidia were viable, whereas 80% of wild type conidia could survive. The *veA*Δ conidia were also more sensitive compared to wild type (Figure 2E). While the *velB*Δ and *vosA*Δ conidia were more sensitive to thermal stress than wild type, the mutant and wild type conidia were equally tolerant to high osmolarity (data not shown). These data indicate that both VelB and VosA are required for trehalose biogenesis in spores, thereby conferring the viability and stress tolerance of spores. The VelB-VosA heterodimer might be the functional unit for these critical biological processes.

### LaeA controls light-dependent formation of the VelB-VosA complex

**Figure 3:**
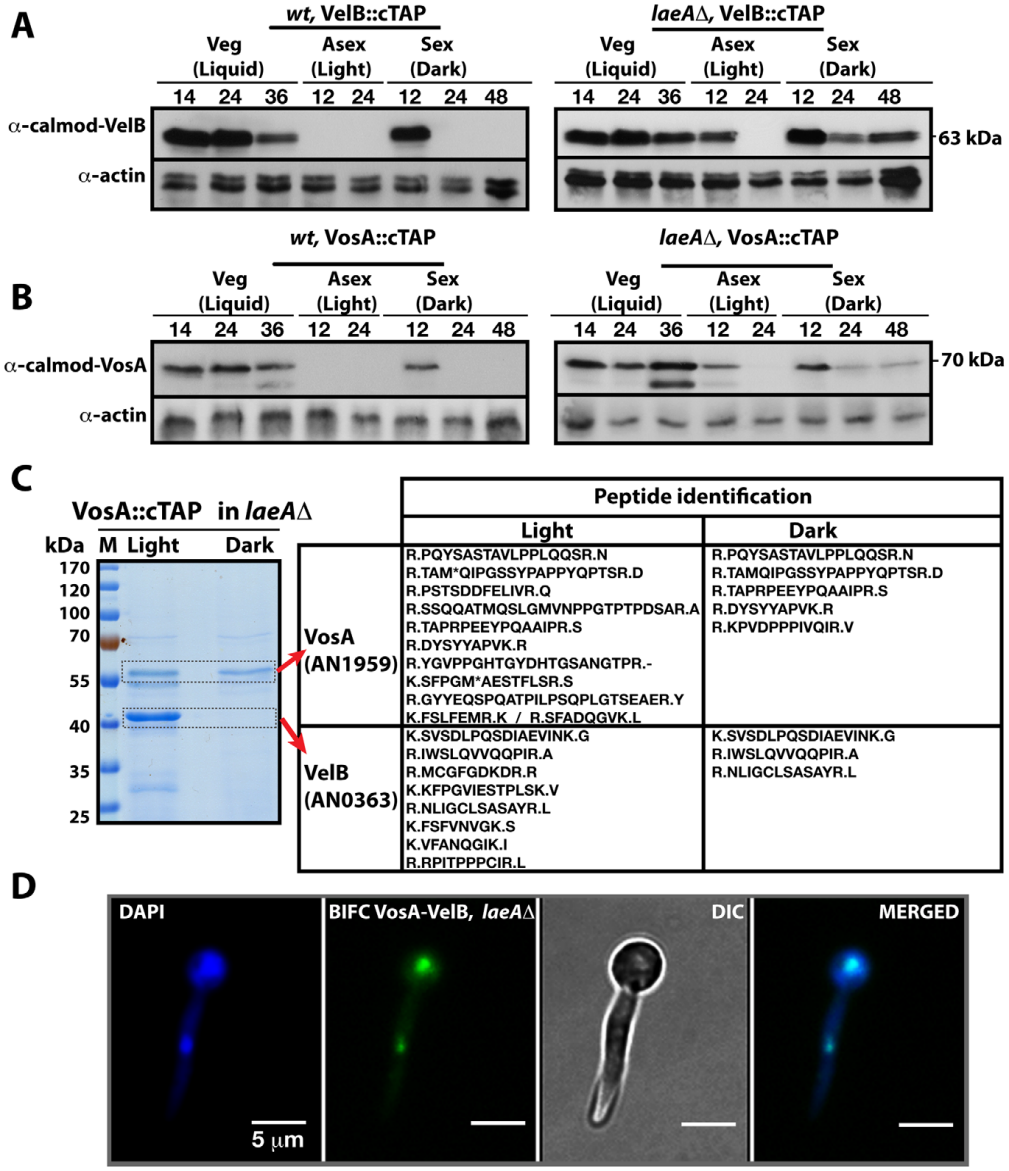
LaeA control of VosA and VelB protein levels and the VosA-VelB complex formation. (A) VelB::cTAP and (B) VosA::cTAP fusion protein levels detected by α-calmodulin antibody during different developmental stages in wild type (*wt*) and *laeA*Δ strains at 37°C. α-actin served as internal control. Protein crude extracts (80 µg) were loaded in each lane. (C) Brilliant blue G-stained 10% SDS-polyacrylamid gel of VosA::cTAP and identified polypeptides (Table S5) in *laeA*Δ strain grown in the light and dark are given. (D) BIFC interaction of the nuclear VosA-VelB complex in *laeA*Δ strain. N-EYFP::VosA interacts with C-EYFP::VelB. Nuclei were counterstained with DAPI (blue).

The finding that both heteromeric complexes are located in the nucleus suggested that there might be a competition for VelB between the nuclear VelB-VeA-LaeA velvet complex and the nuclear VosA-VelB complex. VelB and VosA protein levels were monitored using functional TAP-fusions and the α-calmodulin antibody to address the developmental time window during which both subunits are expressed simultaneously and the VelB-VosA complex can be formed. In wild type cells VelB and VosA are present abundantly during vegetative cultivation in submerged cultures but upon transfer to solid medium in the light both proteins became undetectable. In the dark both proteins were present at the beginning of sexual development (12 h sexual) and then undetectable during later stages of development (Left panels, Figure 3A and 3B). This suggests a potential role of the VosA-VelB complex during vegetative growth and at the beginning of sexual development in the dark when the velvet complex VelB-VeA-LaeA is also present. Simultaneous overexpression of VelB and VosA under an inducible promoter resulted in repression of asexual development, which further supports a common role of both proteins (Figure S1).

We analysed whether the VosA and VelB protein levels depend on VeA or LaeA. Expression analysis in a *veA*Δ strain did not result in significant changes of the VelB or VosA protein levels in comparison to wild type (data not shown). However, in a *laeA*Δ strain, both VosA and VelB were still present after 12 hours incubation in the light. Moreover they also appear during mid sexual stage (24, 48 h sex) (Right panels, Figure 3A and 3B). We performed VosA-TAP purification using a *laeA*Δ strain to determine whether the absence of LaeA also resulted in formation of the VelB-VosA complex in fungal extracts (Figure 3C). TAP purification of VosA from cultures grown in either the light or the dark in the absence of LaeA demonstrated that the VosA-VelB association occured predominantly in the light (Table S5), which is contrary to wild type where we only found the complex in the dark (Figure 1B). Formation of the VosA-VelB nuclear complex in the light in a *laeA*Δ strain was further corroborated by BiFC (Figure 3D). *velB::ctap* and *vosA::ctap* mRNA levels in wild type and *laeA*Δ did not correlate with the protein levels (Figure S2). These results suggest that there is a posttranslational control for the VosA-VelB proteins and LaeA plays a key role in light-dependent control of the VosA and VelB protein levels.

### LaeA controls VeA protein levels and inhibits a molecular size shift from 63 kDa to 72 kDa of VeA

We monitored the cellular levels of the VeA protein during development to explore whether the protein levels of all three members of the velvet family are controlled by LaeA. While it was previously reported that *veA* expression is upregulated in the *laeA*Δ [9], the VeA protein levels have not been analyzed.

**Figure 4:**
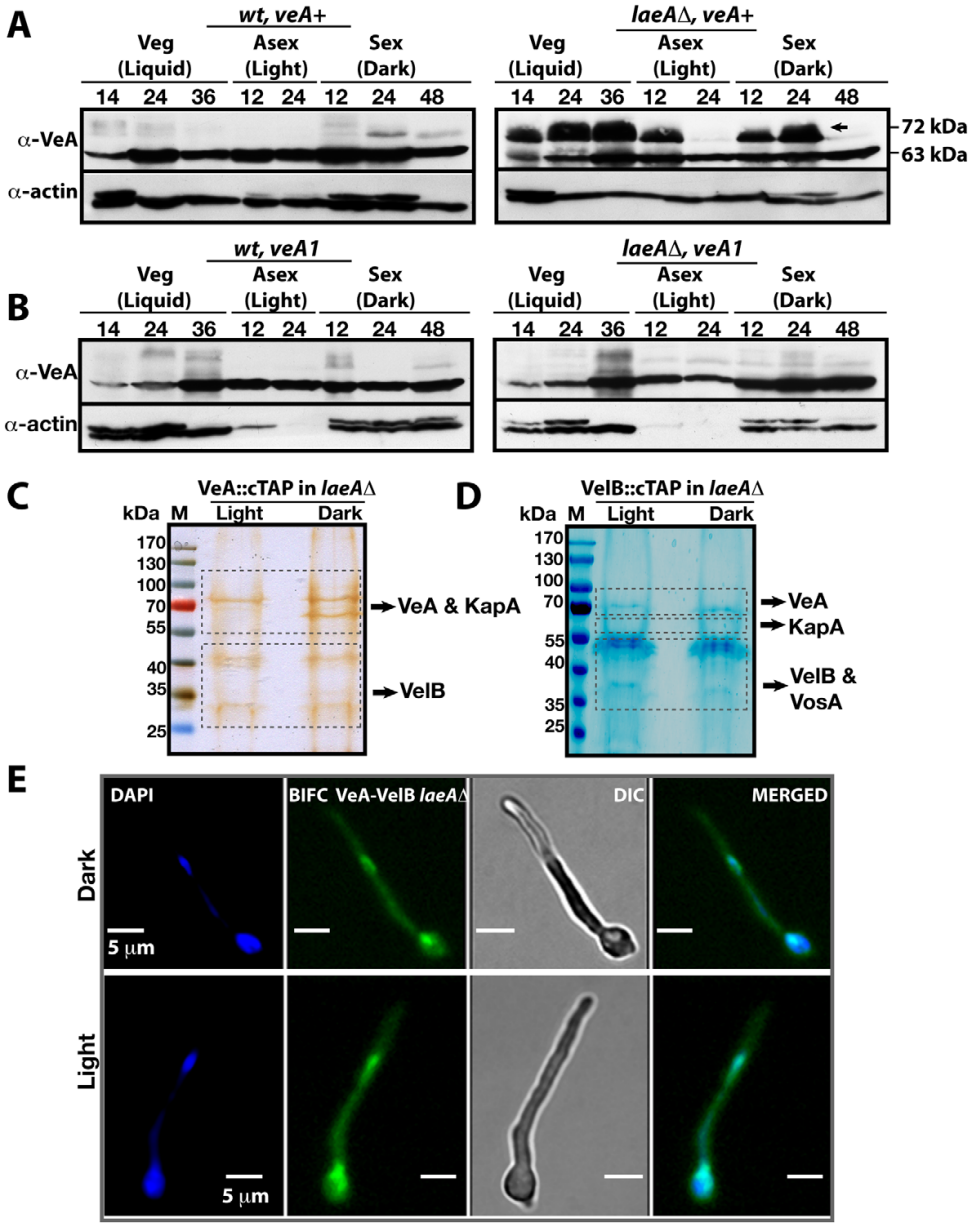
VeA-63 kDa and VeA-72 kDa protein levels in wild type and in *laeA*Δ fungal strains. (A) The VeA protein levels in wild type (*wt*) and *laeA*Δ strains during development (vegetative 14, 24, 36 h in submerged culture, asexual 12, 24 h on plates in the light, sexual 12, 24, and 48 h on plates in the dark at 37°C) by using α-VeA antibodies; α-actin served as internal control. 80 µg total protein was loaded in each lane. (B) The N-terminally truncated VeA1 protein levels in *wt* and *laeA*Δ strains. (C) Silver stained 10% SDS- polyacrylamid gel of VeA::cTAP and identified proteins in *laeA*Δ strain grown in the light and dark (Table S6). (D) SDS-polyacrylamide (10%) gel electrophoresis of VelB::cTAP and associated proteins (in *laeA*Δ *veA*+ strain) stained with brilliant blue G (Table S7). (E) BIFC interactions of N-EYFP::VeA and C-EYFP::VelB in *laeA*Δ fungal cells in light or dark. Nuclei were co-stained by DAPI.

α-VeA antibodies revealed that the cellular levels of the native 63 kDa VeA protein were comparable in wild type and the *laeA*Δ strain in crude cell extracts (Figure 4A and 4B). In addition, a small subpopulation of a VeA isoform of a higher molecular weight (72 kDa) could be detected in wild type cultures during vegetative growth or sexual development in the dark. During the light-mediated asexual development this isoform was hardly detectable. The VeA antibody specifically recognized VeA-63 kDa as well as VeA-72 kDa, because neither bands were present in a *veA*Δ strain (Figure S3A).

This VeA-72 kDa isoform accumulated to higher levels than VeA-63 kDa in the *laeA*Δ strain in vegetative growth and early development with or without light. The total amount of the VeA protein in the absence of LaeA is therefore significantly higher in comparison to wild type. This suggests that LaeA inhibits the overall protein levels of all three members of the velvet family members and specifically inhibits the formation of the 72 kDa VeA isoform.

VeA1 is a peculiar light-insensitive mutant variant of the VeA protein. The *veA1* mutant produces significantly reduced levels of sexual fruiting bodies and constantly high amounts of asexual spores in the dark as well as in the light [23]. The *veA1* mutant phenotype develops by an unknown mechanism and depends on the truncation of the first 36 N-terminal amino acids in comparison to the full-length VeA [24]. This shortened VeA1 mutant protein exhibits reduced protein interaction with VelB and decreased nuclear import of both proteins [9], [25]. In contrast to wild type, the *veA1* mutant did not accumulate VeA-72 kDa (Figure 4B) suggesting that this LaeA dependent molecular shift correlates with light regulation and depends on an intact N-terminal part of VeA. In the presence of VeA1, actin levels decreased presumably due to the increased asexual conidiation (Light 12 and 24), (Figure 4B).

In the absence of LaeA we analyzed complex formation of VeA in the light, when the modified VeA-72 kDa, VosA and VelB proteins accumulated. A VeA::cTAP *laeA*Δ strain was shifted from vegetative liquid growth to solid medium in the light or in the dark for 12 hours to achieve developmental competence. We detected high levels of the VelB-VeA dimer associated with the α-importin KapA under both conditions (Figure 4C and Table S6). The reciprocal experiment using VelB::cTAP recruited VeA and KapA, in addition to VosA. These proteins all co-purified with VelB in the dark as well as in the light (Figure 4C and Table S7). However, VeA::cTAP in wild type recruits these proteins only in the dark, but fails to recruit VosA and only small amounts of VelB in the light [9]. BIFC localization studies revealed that the VeA-VelB interactions in the *laeA*Δ background took place in nuclei of fungal hyphae both in the light and the dark (Figure 4E).

The data suggest that LaeA not only controls the amounts of VosA, VelB and VeA in the light, but also prevents the shift of VeA to the 72 kDa isoform, which presumably represents a post-translational modification. This LaeA controlled VeA modification does not impair the transport of VeA-VelB into the nucleus assisted by the importin KapA. The finding that the importin KapA was only recruited together with VeA(-TAP)-VelB but not with VosA(-TAP)-VelB supports our earlier finding that VelB is preferentially transported into the nucleus together with VeA [9].

### LaeA is required for light-mediated inhibition of sexual development

**Figure 5:**
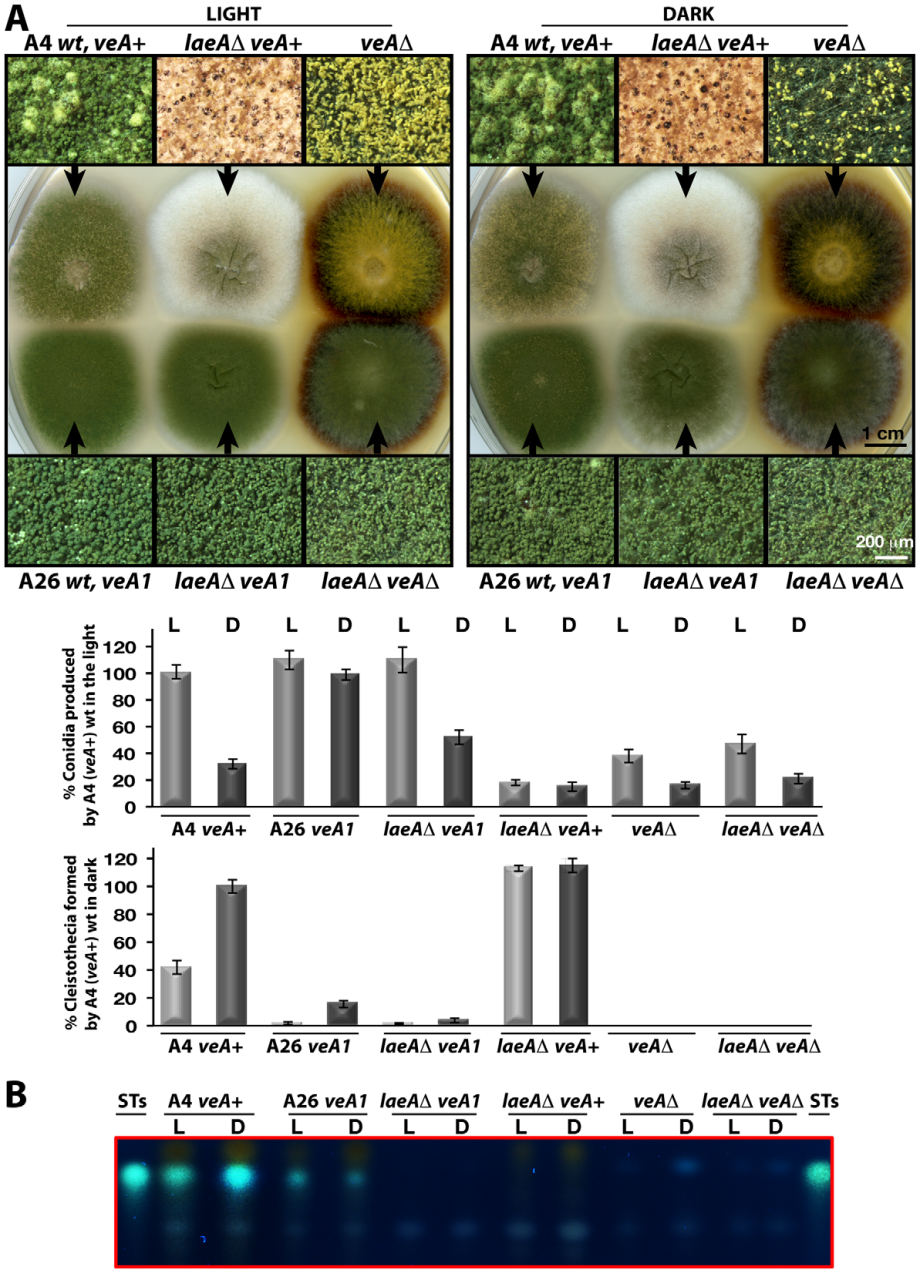
LaeA-VeA as regulators of development and secondary metabolism. (A) Colony morphologies, quantifications of asexual spore (conidia, in light) and fruiting body (cleistothecia, in dark) formations of (A4) *veA*+, (A26) *veA1*, *laeA*Δ/*veA*+, *laeA*Δ/*veA1*, *veA*Δ, *laeA*Δ/*veA*Δ strains grown on the plates at 37°C for 5 days in the light asexually or in the dark sexually. For the quantification of conidia or cleistothecia, the 5×10 mm^2^ sectors from 5 independent plates were used and the standard deviations are indicated as vertical bars. *veA*+ strains conidiation and cleistothecia levels were used as standard (100%). (B) The secondary metabolite sterigmatocystin (ST) production levels of the strains from (A) examined by TLC. 5×10^3^ conidia were point-inoculated at the center of the plates that were kept either in white light (90 µWm^2^) or in dark.

LaeA has been identified as a global regulator of secondary metabolism [15] in light-insensitive *veA1* laboratory strains [24]. The *veA1* allele represents an artificial situation that could be misleading for the understanding of the molecular function of VeA. Therefore we analyzed the *laeA* deletion mutant in the *veA* wild type background, which revealed distinct differences in colony morphology for *veA*+ and *veA1*. The *laeA*Δ *veA*+ colony is white, whereas *laeA*Δ *veA1* exhibits the typical green color of wild type colonies, which is due to the pigmentation of the asexual spores (Figure 5A). All analyzed *laeA*Δ strains irrespective of the *veA* allele were unable to produce the mycotoxin sterigmatocystin (ST) underlining the well-known LaeA function as a global regulator of secondary metabolism (Figure 5B).

Microscopic examination revealed two major differences between the *laeA*Δ *veA*+ strain and the other strains. Wild type as well as *laeA*Δ *veA1* strain produced higher number of conidiophores bearing the asexual spores (conidia) than *laeA*Δ *veA*+ strain in the light and dark. Quantification of the conidia indicated that conidia production in *laeA*Δ in the *veA+* background was significantly decreased in the light to approximately 20% of the wild type and asexual development was unresponsive to illumination (Figure 5A). This suggests that there is a yet unexplored LaeA control for asexual spore formation, which only works in combination with an intact VeA N-terminus.

In addition to a reduced number of conidia, the whitish appearance of *laeA*Δ colonies originated from significantly elevated levels of sexual structures both in the dark and light (Figure 5A). Wild type *veA*+ strain generated few cleistothecia (seen as black or white round structures) and many conidiophore heads (green structures) in the light, but more cleistothecia and less conidiophores in the dark. The *veA1* strain produced only few cleistothecia in the dark, therefore formed predominantly conidia under both light and dark conditions (Figure 5A).

**Figure 6:**
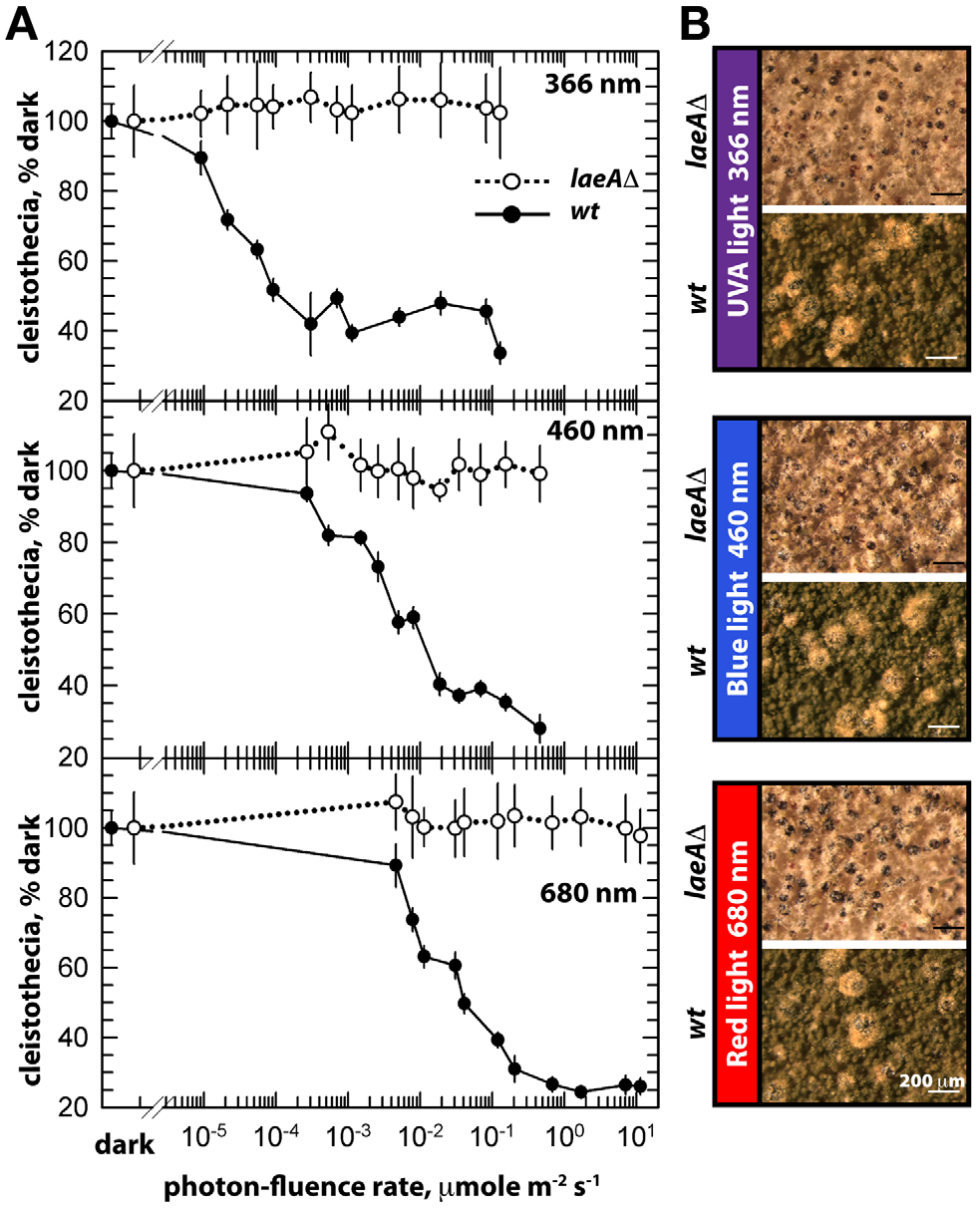
Photon fluence-rate response curves for the photoinhibition of cleistothecia formation in wild type and *laeA*Δ strains. (A) Petri plates point-inoculated with 5×10^3^ spores were irradiated with monochromatic light from overhead position at the given photon-fluence rates. *wt/veA+*; filled circle, *laeA*Δ/*veA+*; open circle. Standard errors are represented by vertical lines. (B) Photographs of fruiting bodies (cleistothecia) of wild type (*wt*) and *laeA*Δ strains under 366-, 460-, and 680_nm_ light illumination.

The unresponsiveness of the *laeA*Δ strain to the white light does not depend on specific light receptors. We determined photon fluence-rate response curves for the photoinhibition of fruiting body formation under near UV for CryA, blue light spectra for LreA-LreB, and red-light spectra for FphA [13], [14]. Wild type strain reduced cleistothecia formation with increasing photo dosage to below 20%. In contrast, the photoinhibition in the *laeA* mutant was lost under all irradiation conditions (UVA _366 nm_, blue _460 nm_, red _680 nm_) (Figure 6). The lack of photoinhibition caused by a loss of LaeA was regardless of high or low light intensity, suggesting that *laeA*Δ strains are entirely blind and LaeA is required for light mediated inhibition of cleistothecia formation of all three known light qualities.

The functional relationship between *laeA* and *veA* was examined by creating the *laeA*Δ *veA*Δ double mutant. The double mutant exclusively manifested the *veA*Δ phenotype characterized by only asexual development. Thus, the *veA* mutation is epistatic to *laeA*Δ and sexual development of *laeA* mutants depends on VeA (Figure 5A). These results demonstrate that LaeA has an additional developmental role besides being a major regulator of secondary metabolism and is an essential part of the light-dependent control mechanism of fungal development. Double mutant strains of *laeA*Δ with *fphA*Δ, *lreA*Δ, *lreB*Δ or *cryA*Δ representing photoreceptor genes always resulted in an epistatic *laeA*Δ phenotype (data not shown). The LaeA dependency of an intact VeA is essential to promote the asexual developmental program and to inhibit the sexual program of *A. nidulans* in the light. Truncation of the N-terminus part of VeA, which interacts with VelB, abolishes this LaeA mediated regulation. This suggests that LaeA controls the protein levels of the members of the regulatory velvet family but also the balance between VelB-VeA, VelB-VeA-LaeA or VosA-VelB complexes within the fungal cell.

### LaeA is part of a cell-specific control for the formation of sex-specific Hülle cells

**Figure 7:**
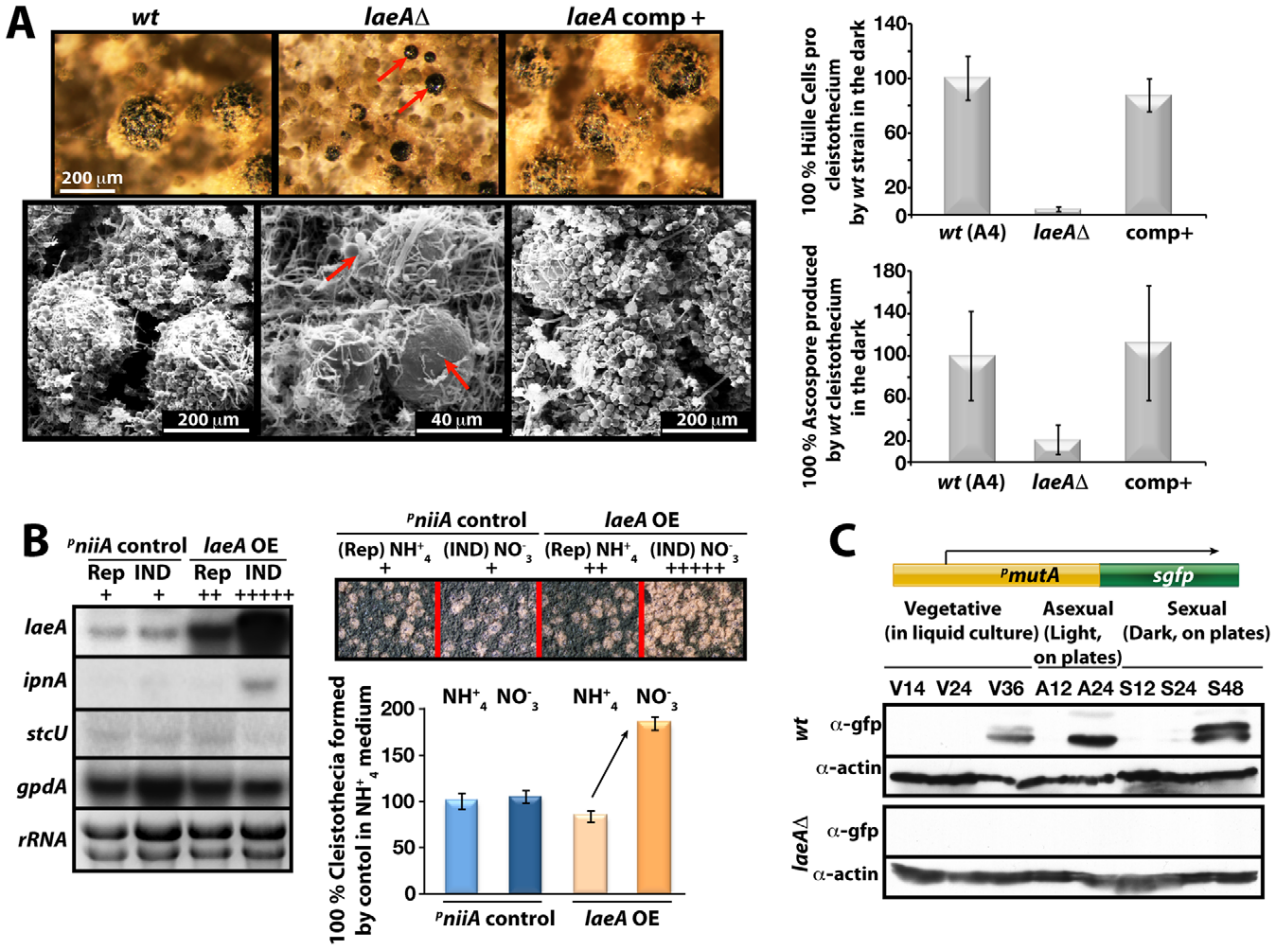
LaeA-dependent Hülle cell formation. (A) Stereo- (top) and scanning electron (SEM) micrographs of wild type (*wt)*, *laeA*Δ, and *laeA* complemented strains and quantification of Hülle cells and ascospores per cleistothecium in the dark. Small cleistothecia produced by *laeA*Δ strain without Hülle cells are indicated by red arrows. Hülle cells and ascospores were counted from 10 different cleistothecia of *wt*, *laeA*Δ and *laeA* complemented strains photographed by SEM. Vertical bars represent standard deviations. Relative values (%) to the numbers of Hülle cells (100–120) or ascospores (2×10^5^) per cleistothecium in wild type are presented. (B) Overproduction of LaeA in *veA*+ strain increases sexual fruiting body formation in the dark. Growth of wild type (*wt*) containing an empty *niiA* promoter plasmid (control), and *^p^niiA::laeA* strains. Repressive (5 mM ammonium tartrate) and inducive (10 mM sodium nitrate) conditions were used to confer different levels of the *niiA* promoter activity. Fruiting body formation of wild type is not affected by these nitrogen sources. The *laeA* transcript levels were monitored by Northern blot analyses in comparison to *ipnA*, *stcU*. *gpdA* levels and ethidium bromide stained rRNA were used as controls; 20 µg RNA were applied in each lane. Spores (5×10^3^) were point-inoculated on solid medium and grown at 37°C for 5 days on plates in the dark and cleistothecia were quantified as described [49]. (C) Western blot analysis of Hülle cell specific activity. *^p^mutA::sgfp* is specifically expressed in Hülle cells. *wt* and *laeA*Δ strains carrying the reporter were grown for indicated time points at 37°C and Western blot with α-gfp, and α-actin as control were performed. 80 µg total protein was applied.

We compared in more detail the constitutively produced fruiting bodies of *laeA*Δ *veA*+ and wild type. This resulted in the discovery of two remarkable phenotypes. Both were verified by complementation of the *laeA*Δ strain by the *laeA* wild type allele (Figure 7A). First, the *laeA*Δ mutant produced more fruiting bodies than wild type but they were significantly smaller in size. Detailed inspection with scanning electron microscope (SEM) unveiled that the wild type fruiting bodies of a diameter of approximately 200 µm were reduced to 40 µm diameter cleistothecia in the *laeA*Δ strain (Figure 7A). In agreement with their small size, cleistothecia of *laeA*Δ contained only 20% of the ascospores compared to wild type fruiting bodies (Figure 7A). The small *laeA*Δ cleistothecia contained meiotically formed viable ascospores which germinated on appropriate medium, indicating that the fertility of ascospores was not affected (data not shown).

Second, wild type cleistothecia are normally covered by spherical Hülle cells forming a tissue which is proposed to nurse the maturing fruiting bodies. In contrast to wild type where cleistothecia were entirely surrounded by hundreds of Hülle cells, the cleistothecia in *laeA*Δ were in contact with only two to five Hülle cells per cleistothecium (Figure 7A).

We examined the influence of various degrees of LaeA overproduction on fungal development for a more comprehensive picture of the LaeA regulatory function in sexual development. We expressed *laeA* under the nitrate inducible *niiA* promoter [26] in the *veA+* backgound (Figure 7B). Induction of *laeA* expression was verified by Northern blot hybridization. The *ipnA* and *stcU* genes were used as control because *ipnA* was previously shown to increase by high levels of LaeA [15] whereas *stcU*, a gene of the ST gene cluster, was not affected. Increasing degrees of LaeA expression did not disturb light inhibition of sexual development which was functional as in wild type (data not shown). Only high levels of LaeA resulted in a significant developmental phenotype in the dark. This overexpression strain produced twice more cleistothecia than wild type, when the *niiA* promoter was activated by cultivation on nitrate medium (Figure 7B). This further corroborates a developmental role of LaeA to control cleistothecia, which might be mediated by the Hülle cells.

Hülle cells were analyzed in more detail by monitoring the expression of cell specific genes in the *laeA*Δ strain. The α-mutanase encoded by *mutA* is particularly expressed in Hülle cells [27]. A *mutA* promoter fusion to *sgfp* (synthetic green fluorescence protein) was constructed in wild type and *laeA*Δ strains. Whereas wild type showed an sGFP signal during late phases of vegetative growth and development, *laeA*Δ strain failed to generate detectable sGFP signal (Figure 7C). The GFP fluorescence of 100 Hülle cells for each strain was measured to analyze whether the single Hülle cell of the *laeA*Δ strain differs from the Hülle cell tissue of wild type. Approximately 35 of the 100 wild type Hülle cells showed a specific sGFP signal originating from the cytoplasm of the Hülle cells (Figure S3C). In contrast, there was hardly any specific sGFP in the Hülle cells of *laeA*Δ strains except for a weak autofluorescence. Transcript analysis of the *mutA* gene in wild type and the *laeA*Δ strains further supported the failure of *laeA* mutants to express the Hülle cell specific *mutA* gene. Regardless of the *veA*+ or *veA1* alleles, the *mutA* mRNA levels were drastically reduced in *laeA*Δ strains in comparison to wild type (Figure S4).

Our data suggest that LaeA affects VeA on gene expression and on protein levels potentially by inhibiting the modification of the VeA-63 kDa protein. The N-terminally truncated VeA1 protein is impaired in this control and also impaired in the interaction with VelB. Consistently, LaeA also controls the cellular levels of VelB and VosA as further members of the VeA regulatory protein family. This regulatory network is involved in the promotion of asexual spore formation in the light (presumably by releasing the repressor function of VosA-VelB) as well as the light-dependent inhibition of sexual development. In addition, LaeA has functions which do not specifically require the VeA N-terminus but require some VeA activity. These include Hülle cell formation and/or controlling the Hülle-cell specific *mutA* gene activity (Figure 7) but also secondary metabolism control including *aflR* expression [15]. These findings predict that there might be more regulatory developmental genes controlled by LaeA either in a VeA N-terminus dependent or independent way.

The screening of transcripts of various fungal developmental regulator genes (Figure S4) revealed that the asexual regulator *abaA* is one of the genes controlled by the LaeA when VeA N-terminus is intact. *abaA* encodes a transcription factor which is conserved from filamentous fungi to yeast [28], [29] and which is required for asexual spore formation. *abaA* expression levels were almost abolished during development of a *veA*+ *laeA*Δ strain. The effect seems to be specific because another key regulator of asexual development, *brlA* [30] was significantly less affected in its expression in the same mutant strains.

Various regulator genes of sexual development exhibited only subtle VeA dependent changes in gene expression during development. The two sexual regulatory genes *nosA* and *steA* [31], [32] were exceptions because they were transiently reduced in the *veA1 laeA* and the *veA+ laeA* deletion strains during vegetative growth (20 h). This effect is therefore independent of the N-terminus of VeA and seems to be specific, because the mRNA for the GATA type transcription factor NsdD, which is essential for sexual development [33], was not significantly changed in wild type in comparison to both *laeA* mutant strains. Indeed, overexpression of *nosA* in *laeA*Δ moderately rescued the small cleistothecia phenotype (Figure S5).

Our data support that LaeA is required not only for differentiation of asexual spores but also for Hülle cells and their activity. It seems plausible that without LaeA and therefore without Hülle cells the cleistothecia are not nursed properly and can not reach their wild type regular size. These results also indicate that formation of the Hülle cells is not an absolute prerequisite for fruiting body formation. Moreover, our results further support that LaeA is involved in the control of regulatory genes in development and secondary metabolism and this control can be dependent or independent of the VeA N-terminus.

## Discussion

### The velvet family of fungal regulatory proteins for cell fate

The velvet family regulatory proteins are fungus-specific and highly conserved among ascomycetes and basidiomycetes [16]. Fungi represent one of the largest groups of eukaryotic organisms on earth with an estimated 1.5 million, mostly unknown, species including human and plant pathogens [34]–[38]. The understanding of the molecular mechanisms of the VeA family proteins function might play a key role to understand fungal development. The VeA family includes VeA, VelB, VelC and VosA. VeA, as the first identified light regulator of this family [23], regulates morphological development coupled with secondary metabolism [10], [17]–[19], [39]. VosA is not only able to repress asexual development in *A. nidulans*, but is also essential to link sporogenesis and trehalose biogenesis [16]. VelB was discovered by its ability to interact with VeA and characterized as a light-dependent developmental regulator [9]. In this study, we also identified the VelB-VosA complex. The appearance of VelB correlates with the VosA protein. VelB and VosA seem to share at least parts of their functions, because overexpression of the dimer represses asexual development and the *velB*Δ strain exhibits similar reduced survival rates as the *vosA* deletion. The genetic data suggest that VelB and VosA are inter-dependent in executing trehalose biogenesis, spore maturation and long-term viability. This may be associated with the formation of the nuclear VelB-VosA heterodimeric complex. Therefore VelB has dual functions within asexual as well as sexual development.

The roles of VelB and VosA in spore maturation are similar to those found in other filamentous fungi including *A. fumigatus* and *Histoplasma capsulatum*. In *H. capsulatum*, Ryp2 and Ryp3, are homologs of VosA and VelB, respectively, and play a role in regulation of sporulation and inter-dependent expression of the *RYP* genes [40]. In *A. fumigatus*, the deletion of *vosA* and *velB* caused ∼50% reduction of the spore trehalose content and viability (Park & Yu, unpublished). Preliminary functional studies of *velC* in *A. nidulans* indicate that this fourth member of the velvet family positively functions in sexual development (Park et al, unpublished).

### The protein complexes: VosA-VelB, VelB-VelB, and VelB-VeA-LaeA

Heteromeric proteins play vital roles in the development of fungi, plants or animals. Fungal examples involved in the development of sex-specific cells include the heterodimeric α2-a1 complex which represses haploid specific gene expression or the α2-MCM1 complex which turns off alpha-specific genes in yeast cells [41]. Combinations of bE (*E*ast) and bW (*W*est) heterodimeric complexes promote the switch from the haploid yeast phase to the pathogenic dikaryotic phase of the corn smut fungus *Ustilago maydis* [42]. Our studies demonstrated that the velvet family proteins form a novel class of fungal regulators that also establish heteromeric complexes and have interdependent functions in determining cell fate.

The VeA-VelB heterodimeric complex of *A. nidulans* presumably forms in the cytoplasm and serves as the major pathway for the VelB entry into the nucleus. The VeA nuclear transport is controlled during development by the light which increases the cytoplasmic fraction of VeA and reduces the nuclear population [25]. The bipartite nuclear localization signal (NLS) is located at the N-terminus of the VeA protein and is disrupted in VeA1, which is derived from a truncation of 36 amino acids of the N-terminus of VeA. This results in the constitutive but reduced VeA nuclear import with reduced interaction with VelB without being controlled by illumination. Light control of VeA might be activated during development by a direct interaction of VeA to the phytochrome FphA. This light sensor is connected to the white collar homolog proteins LreB and LreA as additional light sensors [13]. CryA, another fungal light sensing system, functions in a distinct way. It does not interact with VeA, but reduces *veA* mRNA accumulation and therefore reduces the VeA protein levels within the fungal cell during development [14]. Whereas VelB can form homodimers in both cytoplasm and nucleus, VosA-VelB is preferentially located in the nucleus. If VeA provides the major nuclear import pathway for VelB, this suggests that VeA can be exchanged for VosA or another VelB within the nucleus.

The VosA-VelB heterodimer complex appears to have multiple functions. It can repress asexual spore formation and also controls genes associated with trehalose biogenesis for the spore. The VosA-VelB complex may act as a transcription factor as the C-terminal domain of VosA has transcription activation activity and the VosA protein might bind to the promoter regions of various genes [16]. It will be interesting to reveal the genes regulated by the VosA-VelB complexes among filamentous fungi including human or plant pathogens. While our *in vivo* biochemical studies never identified VelC as an interacting partner of the three velvet regulators, a yeast two hybrid screen followed by GST pull-down assay suggested that VosA and VelC interact and form a heterodimer complex (Ni et al, unpublished data). It appears that *velC* might be expressed at very low levels under specific environmental or developmental conditions.

### LaeA control of VosA and VelB protein levels requires an intact N-terminus of VeA

LaeA fulfills two distinct yet related functions within the fungal cell. One function includes the control of the amount of velvet family proteins and therefore the potential to form various complexes. We found here a specific regulatory role of LaeA for all three velvet family members. This novel regulatory role of LaeA for fungal development exceeds its previously reported function as a global regulator of secondary metabolism [15].

LaeA controls the amount of VosA and VelB in a light dependent manner. In the light the wild type fungus would normally reduce the VosA-VelB complex to release asexual inhibition and to promote the asexual program. In parallel, the sexual program which also requires VelB is repressed. Without LaeA we find, even in the light, high amounts of VosA and VelB and consistent with the VosA-VelB complex, the asexual program is repressed and the sexual pathway is constitutively activated. It is not yet understood why the truncation of the N-terminus of the VeA1 mutant protein results in constitutively high asexual and low sexual development independent of illumination. Activation of sexual development by excessive amounts of the VelB-VosA dimers even under the light conditions further supports that a major function of the VelB-VosA complex after successful germination of spores is to repress fungal development during vegetative growth.

**Figure 8:**
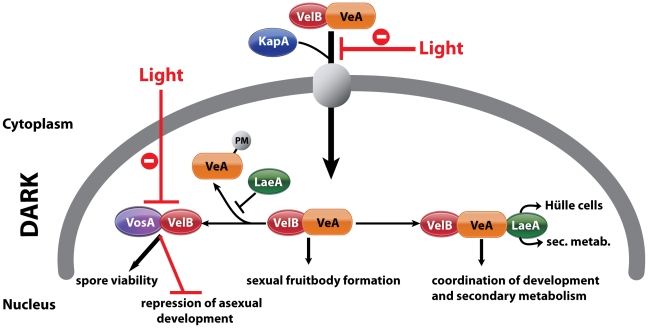
Complexes of velvet family regulatory proteins and LaeA during *A. nidulans* development. This model describes the fungal development in dark and the effect of light on nuclear entry and the formation of VosA/VelB complex. VelB primarily enters the nucleus together with VeA and alpha-importin KapA. Then, VelB can be distributed to two distinct complexes. The VosA-VelB dimer can repress asexual spore formation and controls spore maturation and trehalose biogenesis. VeA-VelB can associate with LaeA and the dimeric and/or the trimeric complex controls sexual development. The association of LaeA with the VelB-VeA complex links the secondary metabolism to the development. LaeA controls Hülle cell formation, secondary metabolism and protects VeA against posttranslational modification (PM). VelB is part of the two complexes, VosA-VelB or VelB-VeA.

LaeA does not only control VosA and VelB protein levels but also controls simultaneously VeA protein levels and the formation of different VeA forms. VeA is constitutively expressed during different phases of fungal development and normally represents a 63 kDa protein. An additional higher molecular weight VeA of 72 kDa is inhibited in the light where asexual development is promoted, and is only detectable during vegetative growth or in the dark during sexual development. The increased amounts of VelB and VosA in the absence of *laeA* somehow correlate with an accumulation of the VeA-72 kDa version. This accumulation can not be observed when the N-terminus is truncated as in the VeA1 mutant protein. The 72 kDa shift from VeA-63 kDa presumably represents a modification which is inhibited by LaeA in a light dependent manner. VeA is known to be a phosphoprotein [13] and phosphatase treatment does not affect VeA-63 kDa or the VeA1 mutant version but resulted in a partial reduction in the mobility of the 72 kDa version (Figure S3B). Furthermore α-phosphoserine and α-phosphothreonine recognized the immunoprecipitated phosphorylated 72 kDa VeA protein in the *laeA*Δ background supporting that the serine and threonine residues of VeA are phosphorylated (data not shown). However, the LaeA dependent VeA modification is even more complex and includes at least one yet unknown modification. LaeA associates with the VelB-VeA dimer forming the heterotrimeric velvet complex. LaeA might protect VeA from modification by occupying the C-terminus of VeA, and thereby controlling the balance between VosA-VelB and VelB-VeA-LaeA (Figure 8). There might be another level of control that limits the overall VeA protein levels. It will be interesting to analyze whether LaeA is able to interfere with the interaction of VeA to the light receptor complex FphA-LreA-LreB [43] to confer its light control function.

### The global regulator of secondary metabolism LaeA is part of the control for Hülle cell formation

Further LaeA regulatory functions are independent of the N-terminus of VeA. It is tempting to speculate that the N-terminus dependent LaeA functions involve VosA and VelB, whereas the independent functions concern LaeA alone or in concert with VeA and/or VelB. The LaeA-VeA1 complex can at least partially fulfill the LaeA control of secondary metabolism, which has been investigated in *veA1* laboratory strains [5], [15].

In a striking contrast to the *veA* and *velB* mutants, loss of LaeA does not abolish the potential to form fruiting bodies. We found it remarkable that without LaeA almost no Hülle cells can be formed, and hardly any expression of the Hülle cell specific *mutA* gene occurs. The function of Hülle cells are proposed to protect and nourish the maturating nests which are the primitive structures of cleistothecia [2]. Consistently to the proposed nursing function, the fungal fruiting bodies of a *laeA* deficient strain are only one fifth of the normal size. The size of an average cleistothecium is around 200 µm. In literature there are few genes affecting the size of cleistothecia including tryptophan auxotrophic mutants [44], *hisB* gene deletion [45] as well as *sumO* mutant. SumO is a small ubiquitin like modifier of *A. nidulans* [46]. The *laeA* deletion mutant constitutively produces these high amounts of small cleistothecia, even in the presence of light, further corroborating the key role that LaeA plays in light dependent fungal development.

Another remarkable finding is that the expression of the transcriptional regulatory genes *steA* [31] and *nosA* are LaeA dependent during vegetative growth. Both genes are involved in the sexual pathway. Without SteA there are no fruiting bodies [31]. Even more interesting is that *nosA* mRNA is completely absent in vegetative cells of *laeA*Δ. Deletion of *nosA* gene also results in very small cleistothecia which are about 30 µm in size but still contain fertile ascospores [32]. *nosA*Δ strain has almost no Hülle cells, a phenotype similar to *laeA*Δ strains. It is therefore likely that LaeA dependent expression of *nosA* during the vegetative stage is required for Hülle cell formation. This is further supported by the findings that overexpression of *nosA* under nitrate inducible *niiA* promoter in *laeA*Δ partially rescued the lack of Hülle cells, small cleistothecia and ascospore production (Figure S5). This results in abundant expression of NosA in vegetative cells in a *laeA* deletion (Figure S5D). The reason why the rescue is only partial might be due to the fact that some other regulators acting in the parallel pathway with *nosA* for Hülle cell formation are still less expressed or misregulated in a *laeA*Δ. It will be interesting to examine whether and how this LaeA dependent temporal control of transcription factor genes like *nosA* depends on the members of the velvet family.

### LaeA: cell-type regulator and master of secondary metabolism

The parental generation of multicellular organisms normally has to provide nourishment as well as protection for the next generation. Hülle cells of the mold *A. nidulans* are associated with cleistothecia and provide this function for the fungal fruiting body. Our major finding here is that LaeA in combination with the velvet family of related regulatory proteins is involved in both lines of support for the next generation. LaeA was first discovered to be the global regulator of secondary metabolite genes including sterigmatocystin, penicillin and many other compounds. All these chemicals might confer a certain advantage to the fungus during growth under substratum in the soil. *Aspergillus* produces asexual conidiation on the surface of the soil, but sexual development takes place under substratum where numerous eukaryotic or prokaryotic organisms compete for nutrients and represent a threat to vulnerable sexual fruiting bodies. Carcinogenic sterigmatocystin might protect fungal cleistothecia against eukaryotic competitors. Consistently, *laeA*Δ strains are the preferred food source of insect larvae in comparison to a wild type strain [6].

Similarly, penicillin might help to defend against various bacteria in the soil. All these responses regulated by LaeA might be considered as the *chemical protection* of fruiting bodies. At the same time, LaeA is essential for the Hülle cells and therefore controls feeding of the fruiting bodies by providing these cells. Thus, LaeA promotes both the production of chemicals to protect fruiting bodies and the production of nourishing cells for developing fruiting bodies.

The LaeA functions exerted on maturating cleistothecia in combination with the heteromeric protein complexes of the velvet family represent an unexpected scenario in fungal development. It will be interesting to see how much convergent evolution there is and whether there are molecular counterparts of LaeA in other higher organisms which are involved in the protective as well as the nutritional function for preparing the next generation for future life.

## Materials and Methods

### Strains, media, and growth conditions

Strains used in this study are listed in Table S1. *Aspergillus nidulans* strains; TNO2A3 (*nkuA*Δ) [47], AGB152 [48], AGB154 [49] served as wild type transformation hosts for the deletion and epitope tagging as well as overexpression experiments. Transformation of the *vosA::ctap* linear construct into AGB152 yielded AGB509 strain. *laeA* deletion cassette containing *ptrA* marker was transformed into TNO2A3 generating *laeA*Δ/*veA1* (AGB468) which was then crossed with AGB154. This crossing gave rise to prototrophic deletion strains *laeA*Δ/*veA1* (AGB512) and *laeA*Δ/*veA+* (AGB493), respectively. AGB493 and AGB509 strains were crossed in order to obtain *vosA::ctap*, *laeA*Δ/*veA*+ combination (AGB510). The *velB::ctap*, *laeA*Δ/*veA*+ hybrid (AGB511) was created by crossing AGB493 with AGB389 strain. The presence of wild type *veA*+ allele was verified by analytical PCR of the locus followed by *BstX*I digestion. *laeA* deletion as well as *vosA*- and *velB*-tap loci were confirmed by Southern blot (Figure S6). AGB513 strain that contains *veA::ctap* in *laeA*Δ strain was created by introducing pME3711 into AGB512. *^p^mutA::sgfp* reporter plasmid, pME3296, was introduced into AGB152 (*wt*) and *laeA*Δ (AGB493) strains yielding AGB514 and AGB515, respectively. The BIFC plasmids, pME3714 (*nyfp::velB*/*cyfp::vosA*), pME3715 (*cyfp::velB/nyfp::vosA*), and pME3717 (*nyfp::velB*/*cyfp::velB*) were introduced into the recipient strain AGB506 yielding AGB516 (*velB*-*vosA*), AGB517 (*vosA*-*velB*), and AGB543 (*velB*-*velB*) BIFC strains, respectively. pME3715 was transformed into *laeA*Δ (AGB468), resulting in AGB544 (*velB*-*vosA, laeA*Δ). *nosA* OE construct (pME3719) was placed in AGB493, which led to AGB545. Integration of the plasmids into the genome was confirmed by diagnostic PCR. DH5α and MACH-1 (Invitrogen) *Escherichia coli* strains were applied for recombinant plasmid DNA. *Aspergillus* and *E. coli* strains were cultured as described previously [14].

### Transformations

Tranformation of *E. coli* and *A. nidulans* was performed as explained in detail [50], [51].

### Construction of linear and circular recombinant DNA

During processing and construction of linear and circular DNAs, standard recombinant DNA technology protocols were followed as given in detail [52]. Plasmids and oligonucleotides (Invitrogen) employed in the course of this study are listed in Table S2 and Table S3, respectively. PCR reactions [53] were performed with various DNA polymerase combinations including *Pfu* (MBI Fermentas), *Phusion* (Finnzymes), *Platinum*-*Taq* (Invitrogen) and *Taq* polymerases.

### Generation of linear *laeA*Δ cassette and construction of *laeA* complementation and overexpression plasmids

In order to create *laeA* deletion construct 5′ UTR region of *laeA* was amplified from the wild type genomic DNA with primers OSB22/24 and 3′ UTR region was amplified with OSB25/27. The two amplicons were fused to the *ptrA* marker (from pPTRII) with fusion PCR [47] (nested oligos OSB23/26) yielding 4324 bp linear deletion construct which was used to transform TNO2A3 to AGB468. For complementation of *laeA*Δ, the *laeA* genomic locus (3.7 kb), containing 1.5 kb promoter and 1 kb terminator regions, was amplified from genomic DNA (OSB22/27) and cloned into the *Stu*I site of pAN8-1 (*phleo* ^R^) which yielded pME3635. Then pME3635 was introduced into *laeA*Δ strains, (*veA*+, AGB493) and (*veA1*, AGB512), resulting in AGB494 and AGB518, respectively. In order to overexpress *laeA* gene, *laeA* cDNA was amplified from cDNA library (OZG61/62) and inserted into the *Pme*I site (pME3718) under nitrogen source regulable *niiA* promoter, generating pME3716. This plasmid was eventually introduced into AGB152, which resulted in AGB519.

### Generation of linear *vosA::ctap* gene replacement fragment

To replace the *vosA* locus with *vosA::ctap*, *vosA* ORF including 1 kb of the *vosA* promoter (oligos VosA–A/C) and 1 kb *vosA* terminator (VosA–D/F) were amplified from genomic DNA and the resulting amplicons were fused to the *ctap::natR* module via fusion PCR (VosA–B/E). Gene replacement cassette was introduced into AGB152 and the substitution of the *vosA* locus by *vosA::ctap* was verified by Southern blot hybridization (Figure S6).

### Construction of the BIFC and *nosA* overexpression plasmids

*velB* cDNA was amplified (OZG397/64 for *n-yfp*, OZG63/64 for *c-yfp* fusion) from sexual cDNA library. Then *n-* (OZG73/387) and *c-yfp* (OZG75/77) amplicons were fused to *velB* cDNAs with oligos OZG397/64 (*n-yfp::velB*) and OZG63/64 (*c-yfp::velB*), respectively. *n-yfp::velB* and *c-yfp::velB* were cloned into the *Pme*I site of pME3160 yielding plasmids pME3712 and 3713, respectively. *vosA* cDNA was also amplified (OZG436/438 for *n-yfp*, OZG437/438 for *c-yfp* fusion) from sexual cDNA library. *vosA* cDNA amplicons (OZG436/438) and (OZG437/438) were fused to *n-yfp* (OZG73/387) and *c-yfp* (OZG75/388) via fusion PCR [54]. *c-yfp::vosA* and *n-yfp::vosA* fragments were inserted into *Swa*I site of pME3712 and 3713, respectively. Plasmids bearing *n-yfp::velB*/*c-yfp::vosA* and *c-yfp::velB*/*n-yfp::vosA* were named as pME3714 and pME3715, respectively. For the analysis of VelB-VelB dimer formation, *c-yfp::velB* fragment was cloned into the *Swa*I site of pME3712 generating pME3717. *nosA* cDNA, which was amplified from sexual cDNA library (OZG320/321), was cloned into the *Pme*I site of pME3718 yielding pME3719.

### Construction of the *veA::ctap/natR* plasmid

The *veA::ctap* fusion construct encompassing the promoter and terminator sequences was amplified from pME3157 with oligos OZG304/305. This amplicon was cloned in the blunted *Apa*I site of pNV1 [55] generating pME3711.

### Hybridization techniques and analysis of nucleic acids

Northern [56] and Southern [57] hybridization experiments were performed as given in detail [9]. Band densities in the Northern blots were analyzed with IMAGEJ (National Institutes of Health) and normalized against rRNA. DNA and amino acid sequences were analyzed by using Lasergene software (DNAstar). Northern blot probes were generated by PCR amplification of the following genes (primer sets): *abaA* cDNA (*abaA5*/*abaA3*), *brlA* cDNA (*brlA5*/*brlA3*), *mutA* cDNA (*mutA5*/*mutA3*), *nosA* cDNA (*nosA5*/*nosA3*), *steA* gDNA (*steA5*/*steA3*), *nsdD* cDNA (*nsdD5*/*nsdD3*), *aflR* gDNA (*aflR5*/*aflR3*), *laeA* cDNA (OZG61/OZG62), *gpdA* gDNA (*gpdA5*/*gpdA3*), *tpsA* gDNA (OMN176/OMN177), *orlA* gDNA (OMN182/OMN183), *ipnA* gDNA (*ipnA5*/*ipnA3*), and *stcU* gDNA (*stcU5*/*stcU3*).

### Spore viability test

Viability of spores was examined as described [16]. Two-day old conidia (10^5^ per plate) of wild type and the mutants were spread on solid minimal medium (MM) and incubated at 37°C. After 2∼10 days the conidia were collected and counted in a hemocytometer. Approximately 200 conidia were inoculated on solid MM and incubated for 2 days at 37°C. Survival rates were calculated as a ratio of the number of growing colonies to the number of spores inoculated. This test was performed in triplicate.

### Trehalose assay

Trehalose was extracted from conidia and analyzed as described previously [16], [58]. Two-day old conidia (2×10^8^) were collected and washed with ddH_2_O. Conidia were resuspended in 200 µl of ddH_2_O and incubated at 95°C for 20 min and the supernatant was collected by centrifugation. The supernatant was mixed with equal volume of 0.2 M sodium citrate (pH 5.5) and samples were incubated at 37°C for 8 h with or without 3 mU of trehalase (Sigma), which hydrolyzes trehalose to glucose. The amount of glucose generated was assayed with a glucose assay kit (Sigma). The amount of glucose by deducting trehalase untreated sample from trehalase-treated sample was converted into the trehalose amount (pg) per conidium (triplicate).

### Stress tolerance test

Oxidative stress tolerance test was carried out as described previously [59]. Hydrogen peroxide sensitivity of conidia was tested by incubating 1 ml of conidial suspensions containing 10^5^ conidia with varying concentrations (0.0, 0.25 or 0.5 M) of H_2_O_2_ for 30 min at RT. Each conidia suspension was then diluted with ddH_2_O, and the conidia were inoculated into solid MM. After incubation at 37°C for 48 h, colony numbers were counted and calculated as a ratio to the untreated control. Sensitivity to oxidative stress was also tested by spotting 10 µl of serially diluted conidia (10 to 10^5^) on solid MM with 0, 2.5, 5 M of H_2_O_2_ and incubated at 37°C for 48 h.

UV tolerance test was carried out as described previously [60] with a slight modification. Two-day old conidia were collected in ddH_2_O and plated out on solid MM (100 conidia per plate). The plates were then irradiated immediately with UV using a UV crosslinker and the plates were further incubated at 37°C for 48 h. The colony numbers were counted and calculated as a ratio to the untreated control. UV sensitivity was also tested by spotting 10 µl of serially diluted conidia (10 to 10^5^) on solid MM, which were then irradiated with UV and incubated at 37°C for 48 h.

### Immunoblotting

For detection of GFP signal in 80 µg protein extracts, α-gfp mouse antibody (SantaCruz) was used in combination with One-Hour Western kit (Genscript). α-Calmodulin rabbit antibodies (Millipore) in 1∶1000 dilution in TBS 5% (w/v) non fat dry milk and secondary goat α-rabbit antibodies 1∶1000 in dilution in TBS 5% (w/v) milk were used for the recognition of TAP tag fusion proteins in 80 µg protein extracts. Polyclonal α-VeA antibody recognizing the native VeA protein was raised in rabbit (Genscript). α-VeA antibody (5 µg) in TBST 5% (w/v) milk 0.2% (v/v) Tween-20 was used for the detection of the VeA protein in 80 µg protein extracts in immunoblotting.

### Dephosphorylation assay

Protein extracts were prepared in B buffer (100 mM Tris pH 7.5, 300 mM NaCl, 10% Glycerol, 0,1% NP-40, 1 mM DTT, protease inhibitor mix (Roche)) without phosphatase inhibitors. Total protein extract (1 mg) was treated with 10 units of Shrimp Alkaline Phosphatase (SAP, MBI Fermentas) at 37°C for 30 min. SAP-treated extracts were used for immunoblotting.

### Tandem Affinity Purification (TAP) protocol

Tap tag experiments and preparation of the protein crude extracts were performed as explained in detail [9].

### LC-MS/MS protein identification

Protocols given elsewhere [9] were followed for further data processing and analysis of the proteins.

### Fluorescence microscopy

*A. nidulans* spores (2000) were inoculated in 8 chambered borosilicate coverglass system (Nunc) supplemented with liquid medium. Fluorescence photographs were taken with an Axiovert Observer. Z1 (Zeiss) microscope equipped with a QuantEM:512SC (Photometrics) digital camera and the SlideBook 5.0 software package (Intelligent Imaging Innovations). For BIFC and GFP studies the following parameters were used; YFP filter 1000 milliseconds (ms), RFP filter 600 ms, DAPI filter 40 ms, DIC filter 200 ms, and GFP filter 400 ms.

### Sterigmatocystin (ST) and Thin Layer Chromatography (TLC) analysis

Extraction of ST and running on TLC plates were performed as described in detail elsewhere previously [49].

## Supporting Information

Figure S1 The VosA-VelB dimer and fungal development. Overexpression of *vosA*-*velB* under nitrate inducible bidirectional *niiA*/*niiD* promoter. (A) Asexual development of control strain (empty *niiA*/*niiD* plasmid), and *vosA*-*velB* OE strain (*^p^niiD::nyfp::vosA*-*^p^niiA::cyfp::velB*) on either ammonium (repressive) or nitrate (inducing) containing plates as nitrogen source under light at 37 ^o^C for 3 days. (B) Quantification of asexual conidiation from plates (A). 5x10^3^ conidia were point inoculated. From three independent plates, three sectors (10 mm^2^) were counted and asexual conidiation of the control strain was used as 100% standard. Calculated standard deviations are indicated as vertical bars.
(2.29 MB TIF)

Figure S2 Transcript levels of *velB::ctap* and *vosA::ctap* during different developmental stages in wild type and *laeA*Δ strain. (A) Expression of *velB::ctap* in the wild type and *laeA*Δ strain during vegetative growth (14, 24, and 36 hours), after post asexual induction under light (12, 24 hours), and sexual induction in the dark (12, 24, and 48 hours). (B) Expression studies with *vosA::ctap* fusion at the same time points of development. *gpdA* gene expression and ethidium bromide stained rRNA were used as loading controls. 20 µg RNA was used for each lane.
(1.05 MB TIF)

Figure S3 Hyperphosphorylation of VeA and *^p^mutA* driven GFP signal in Hülle cells in wild type and *laeA*Δ. (A) α-VeA antibody specifically recognizes two VeA protein bands in *laeA*Δ/*veA*+ (*laeA*Δ); *veA*Δ strain (14 h vegetative) as control. (B) Hyperphosphorylation and posttranslational modification of VeA proteins in *laeA*Δ/*veA*+ from 14 and 24 h of vegetative growth. +Ph; Phosphatase treatment, -Ph; No phosphatase treatment. 80 µg total protein was used for both immunoblots. (C) Comparison of Hülle cell specific and autofluorescence activity of GFP signal in Hülle cells. Analysis is based on GFP reporter signal expressed by *mutA* promoter in wild type and *laeA*Δ. Hülle cells were separated from the cleistothecia by vortexing. n:100 Hülle cells from wild type and *laeA*Δ strain were analyzed under fluorescence microscope. Strong real GFP signal originates from the cytoplasm of the Hülle cells and autofluorescence signal stems from the whole body of Hülle cells including thick round cell wall.
(0.74 MB TIF)

Figure S4 LaeA-dependent gene expression. Developmental Northern hybridizations performed in *wt* (*veA*+), *laeA*Δ/*veA1* (results in N-terminal truncation of the VeA protein), *laeA*Δ/*veA+* strains. Fungal strains were grown in submerged cultures vegetatively for 20 h, on plates asexually (in the light) for 6, 12, and 24 h and on plates sexually for 12 & 24 (in the dark). Total RNA was isolated and transcript levels of genes encoding various regulators of development were monitored. The glycolytic gene *gpdA* levels served as internal expression control and ethidium bromide-stained ribosomal RNA (rRNA) was used as loading control. 20 µg total rRNA was used for each stage.
(1.32 MB TIF)

Figure S5 *nosA* overexpression in *laeA*Δ. Partial rescue of Hülle cell and ascospore formation combined with increased cleistothecia size (A) Stereomicroscope pictures of wild type (*wt*), *laeA*Δ, and *nosA* OE strains. (B) Determination of the number of protective Hülle cells. Vertical bars represent standard deviations. The wild type Hülle cell production serves as standard (100%). (C) Quantification of the meiotically produced sexual ascospores. 10 independent cleistothecia were isolated and ascospores were counted. (D) Verification of *nosA* overexpression and monitoring *laeA* expression in *wt*, *laeA*Δ, and *nosA* OE *laeA*Δ by Northern hybridization. *gpdA* expression and ethidium bromide-stained rRNA served as loading control. Strains were grown vegetatively (20 hours) and 20 µg RNA was loaded in each lane.
(2.09 MB TIF)

Figure S6 Southern hybridizations to verify the fungal strains constructed. (A). Comparative genomic architectures of the *laeA* (AN0807.3) and *laeA* deletion loci. The black bar indicates the region encompassed by Southern hybridization. (B) Autoradiography results of Southern hybridization verify the homologous gene replacement in the *laeA* locus for strains *laeA*Δ, *vosA::ctap* in *laeA*Δ, *velB::ctap* in *laeA*Δ. Sizes of the detected restriction fragments are in agreement with the theoretical maps of the loci (A). The numbers at the bottom of the autoradiographs represent the size of the restriction fragments released as base pairs. (C) Relative illustrations of the *vosA* (AN1959.3) and *vosA::ctap* loci. The black bar indicates the region used for the Southern probe. (D) Autoradiographies of *vosA::ctap* in *wt*, *vosA::ctap* in *laeA*Δ, and *velB::ctap* in *laeA*Δ. Restriction bands confirm the loci maps (C).
(1.58 MB TIF)

Table S1 Fungal strains used in this study.
(0.09 MB DOC)

Table S2 Plasmids employed in this study.
(0.06 MB DOC)

Table S3 Oligonucleotides utilized for plasmid constructions and northern hybridizations.
(0.09 MB DOC)

Table S4 SEQUEST Multiple Consensus Report of VosA::cTAP tag identifications after nano-LC-ESI-MS2.
(0.06 MB DOC)

Table S5 SEQUEST Multiple Consensus Report of VosA::cTAP tag identifications in *laeA*Δ after nano-LC-ESI-MS2.
(0.09 MB DOC)

Table S6 SEQUEST Multiple Consensus Report of VeA::cTAP tag identifications in *laeA*Δ after nano-LC-ESI-MS2.
(0.19 MB DOC)

Table S7 SEQUEST Multiple Consensus Report of VelB::cTAP tag identifications in *laeA*Δ after nano-LC-ESI-MS2.
(0.19 MB DOC)
