# Supplementary figures and images for: LaeA Control of Velvet Family Regulatory Proteins for Light-Dependent Development and Fungal Cell-Type Specificity
Source: PLoS Genet. 2010 Dec 2;6(12):e1001226. doi: 10.1371/journal.pgen.1001226 (PMC2996326; doi:10.1371/journal.pgen.1001226)

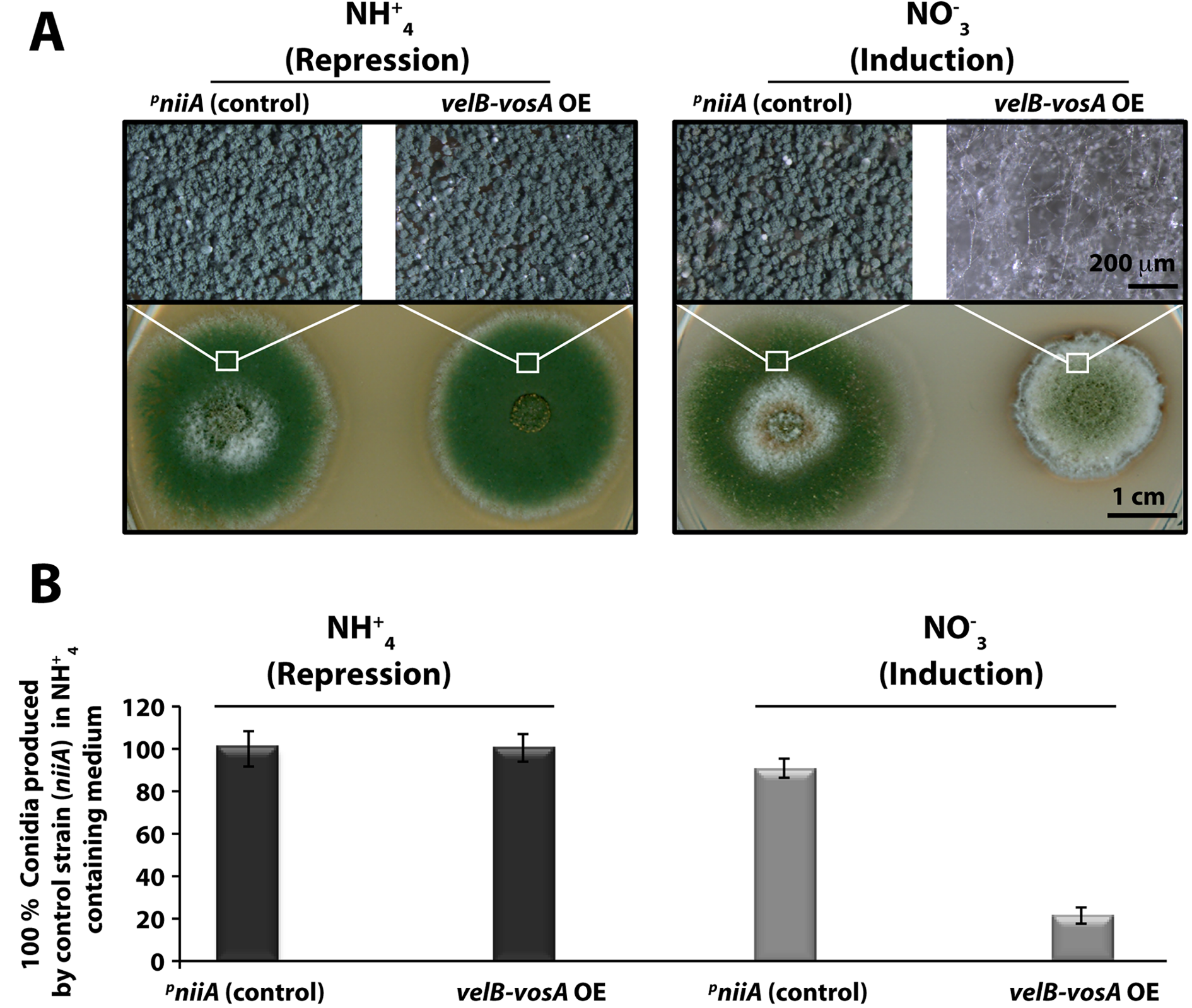

Supplement: Figure S1 — The VosA-VelB dimer and fungal development. Overexpression of vosA-velB under nitrate inducible bidirectional niiA/niiD promoter. (A) Asexual development of control strain (empty niiA/niiD plasmid), and vosA-velB OE strain (pniiD::nyfp::vosA-pniiA::cyfp::velB) on either ammonium (repressive) or nitrate (inducing) containing plates as nitrogen source under light at 37 oC for 3 days. (B) Quantification of asexual conidiation from plates (A). 5x10^3^ conidia were point inoculated. From three independent plates, three sectors (10 mm2) were counted and asexual conidiation of the control strain was used as 100% standard. Calculated standard deviations are indicated as vertical bars. (2.29 MB TIF) [file pgen.1001226.s001.tif]

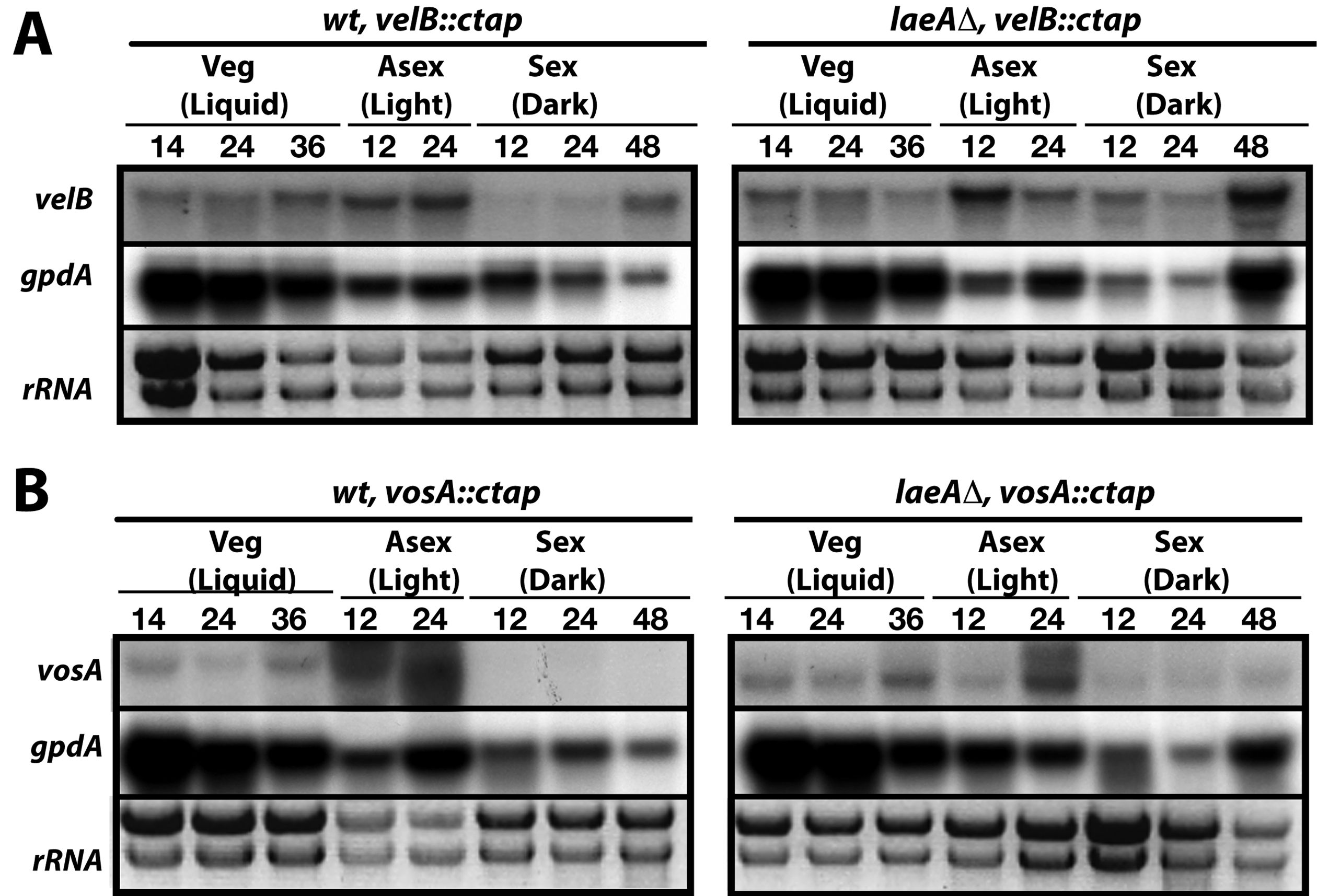

Supplement: Figure S2 — Transcript levels of velB::ctap and vosA::ctap during different developmental stages in wild type and laeAΔ strain. (A) Expression of velB::ctap in the wild type and laeAΔ strain during vegetative growth (14, 24, and 36 hours), after post asexual induction under light (12, 24 hours), and sexual induction in the dark (12, 24, and 48 hours). (B) Expression studies with vosA::ctap fusion at the same time points of development. gpdA gene expression and ethidium bromide stained rRNA were used as loading controls. 20 µg RNA was used for each lane. (1.05 MB TIF) [file pgen.1001226.s002.tif]

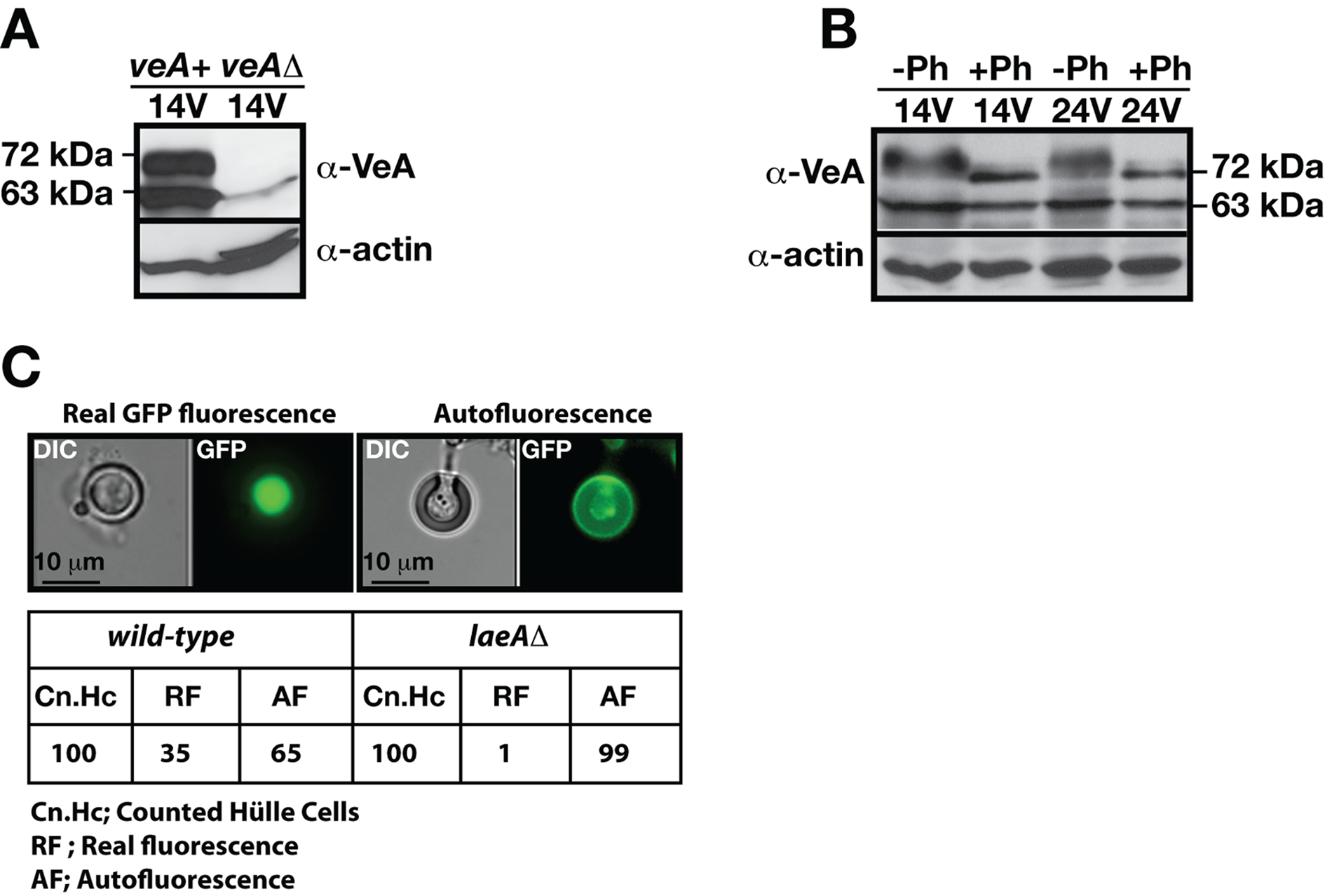

Supplement: Figure S3 — Hyperphosphorylation of VeA and pmutA driven GFP signal in Hülle cells in wild type and laeAΔ. (A) α-VeA antibody specifically recognizes two VeA protein bands in laeAΔ/veA+ (laeAΔ); veAΔ strain (14 h vegetative) as control. (B) Hyperphosphorylation and posttranslational modification of VeA proteins in laeAΔ/veA+ from 14 and 24 h of vegetative growth. +Ph; Phosphatase treatment, -Ph; No phosphatase treatment. 80 µg total protein was used for both immunoblots. (C) Comparison of Hülle cell specific and autofluorescence activity of GFP signal in Hülle cells. Analysis is based on GFP reporter signal expressed by mutA promoter in wild type and laeAΔ. Hülle cells were separated from the cleistothecia by vortexing. n:100 Hülle cells from wild type and laeAΔ strain were analyzed under fluorescence microscope. Strong real GFP signal originates from the cytoplasm of the Hülle cells and autofluorescence signal stems from the whole body of Hülle cells including thick round cell wall. (0.74 MB TIF) [file pgen.1001226.s003.tif]

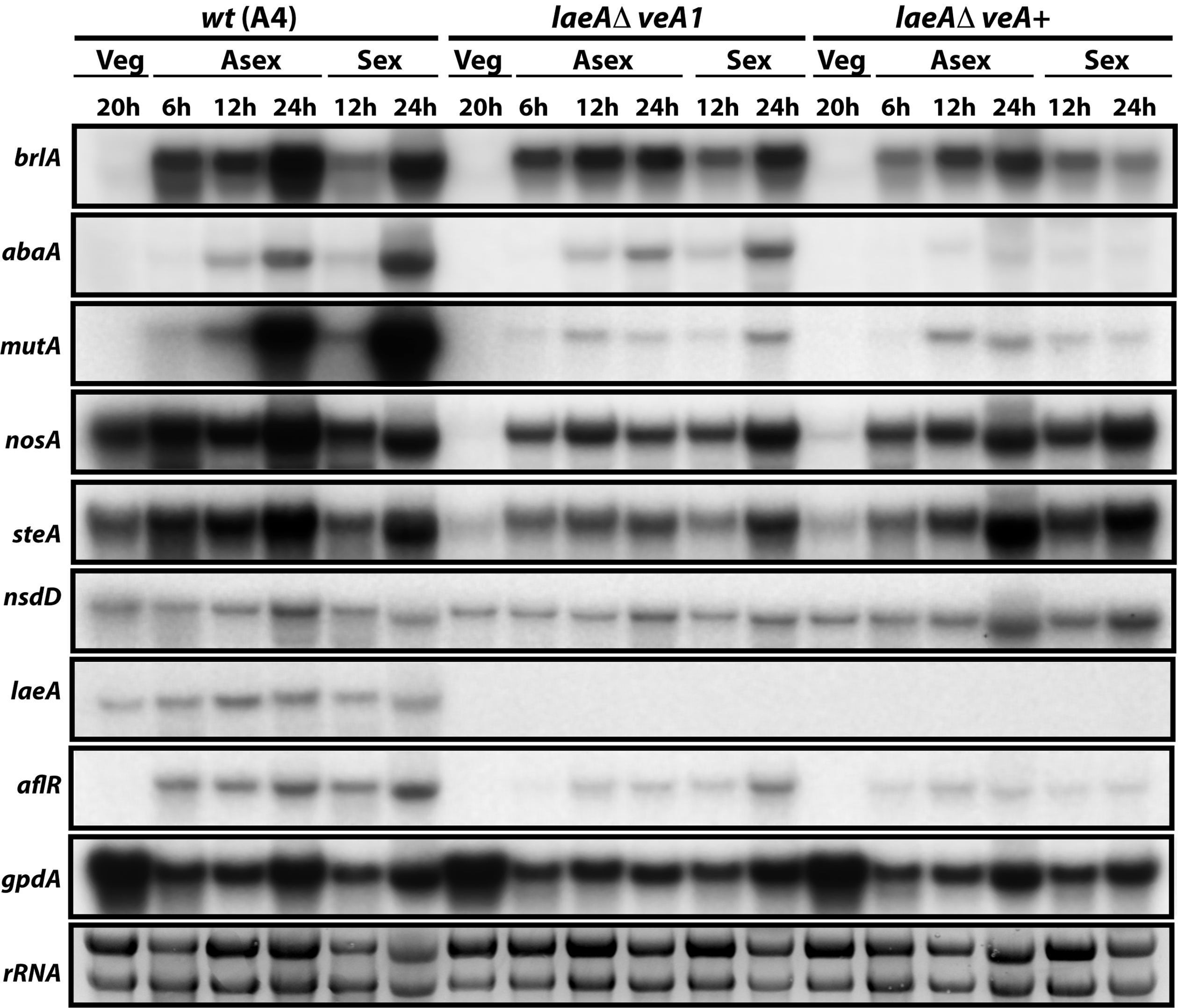

Supplement: Figure S4 — LaeA-dependent gene expression. Developmental Northern hybridizations performed in wt (veA+), laeAΔ/veA1 (results in N-terminal truncation of the VeA protein), laeAΔ/veA+ strains. Fungal strains were grown in submerged cultures vegetatively for 20 h, on plates asexually (in the light) for 6, 12, and 24 h and on plates sexually for 12 & 24 (in the dark). Total RNA was isolated and transcript levels of genes encoding various regulators of development were monitored. The glycolytic gene gpdA levels served as internal expression control and ethidium bromide-stained ribosomal RNA (rRNA) was used as loading control. 20 µg total rRNA was used for each stage. (1.32 MB TIF) [file pgen.1001226.s004.tif]

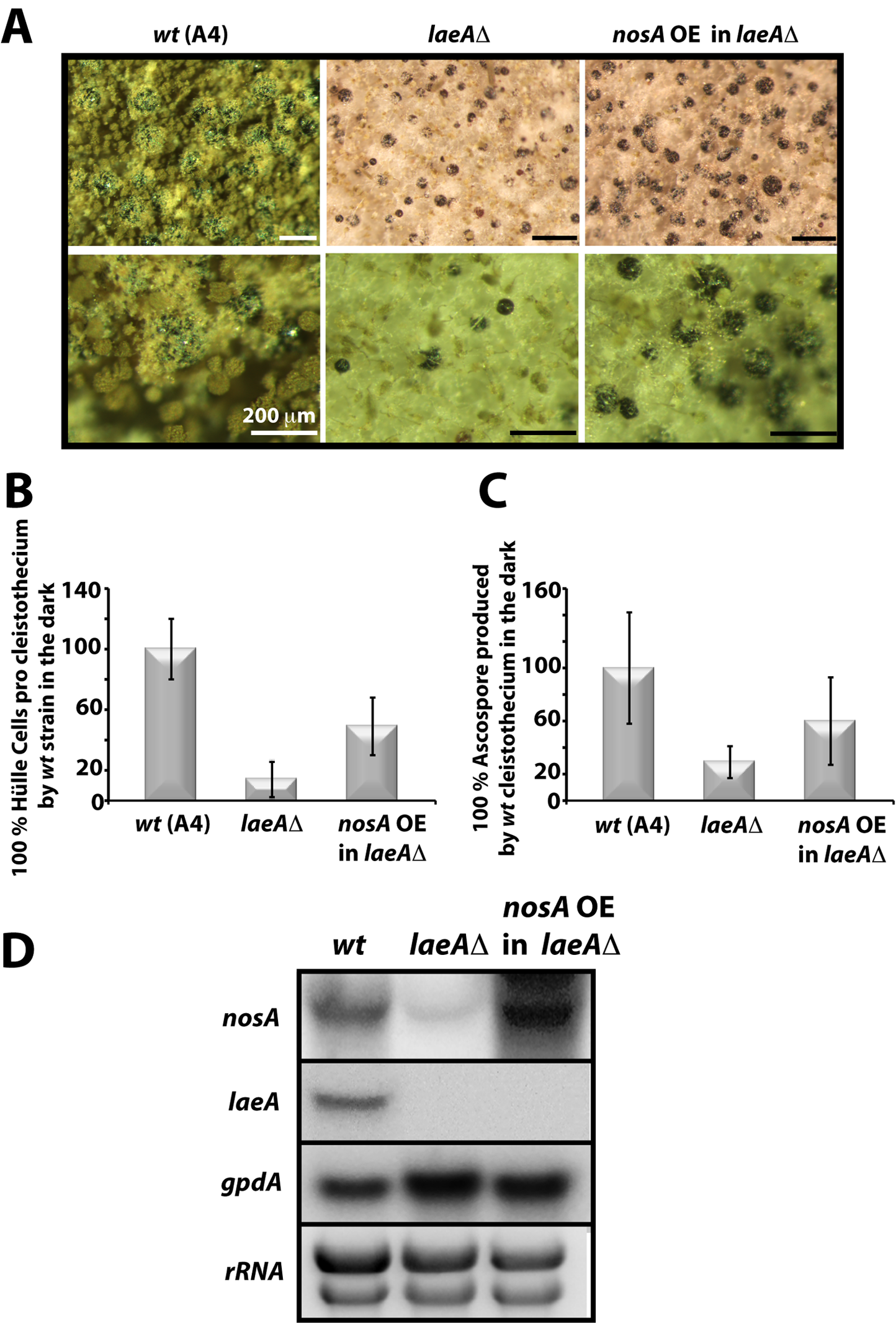

Supplement: Figure S5 — nosA overexpression in laeAΔ. Partial rescue of Hülle cell and ascospore formation combined with increased cleistothecia size (A) Stereomicroscope pictures of wild type (wt), laeAΔ, and nosA OE strains. (B) Determination of the number of protective Hülle cells. Vertical bars represent standard deviations. The wild type Hülle cell production serves as standard (100%). (C) Quantification of the meiotically produced sexual ascospores. 10 independent cleistothecia were isolated and ascospores were counted. (D) Verification of nosA overexpression and monitoring laeA expression in wt, laeAΔ, and nosA OE laeAΔ by Northern hybridization. gpdA expression and ethidium bromide-stained rRNA served as loading control. Strains were grown vegetatively (20 hours) and 20 µg RNA was loaded in each lane. (2.09 MB TIF) [file pgen.1001226.s005.tif]

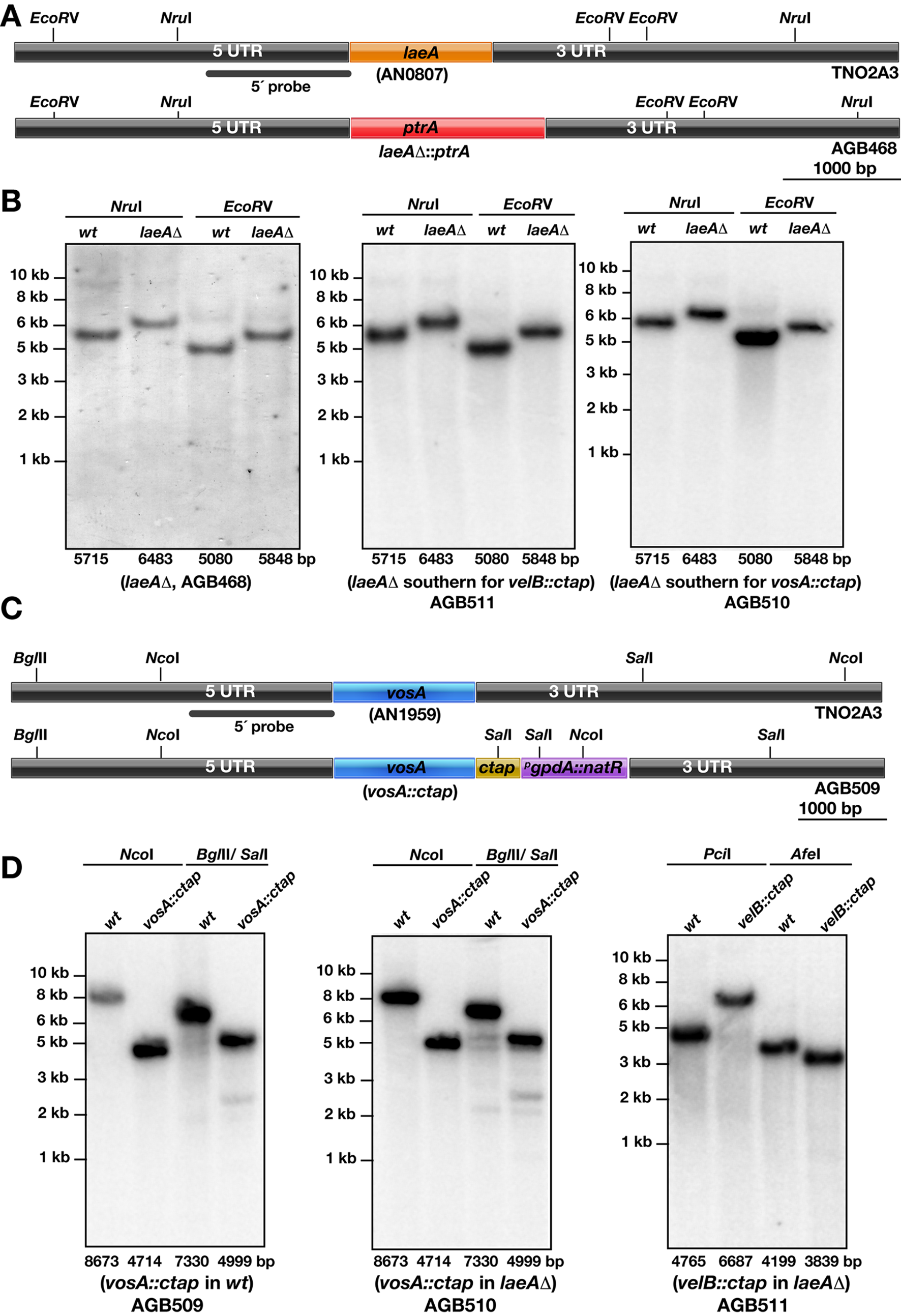

Supplement: Figure S6 — Southern hybridizations to verify the fungal strains constructed. (A). Comparative genomic architectures of the laeA (AN0807.3) and laeA deletion loci. The black bar indicates the region encompassed by Southern hybridization. (B) Autoradiography results of Southern hybridization verify the homologous gene replacement in the laeA locus for strains laeAΔ, vosA::ctap in laeAΔ, velB::ctap in laeAΔ. Sizes of the detected restriction fragments are in agreement with the theoretical maps of the loci (A). The numbers at the bottom of the autoradiographs represent the size of the restriction fragments released as base pairs. (C) Relative illustrations of the vosA (AN1959.3) and vosA::ctap loci. The black bar indicates the region used for the Southern probe. (D) Autoradiographies of vosA::ctap in wt, vosA::ctap in laeAΔ, and velB::ctap in laeAΔ. Restriction bands confirm the loci maps (C). (1.58 MB TIF) [file pgen.1001226.s006.tif]
