# Supplementary material for: LaeA Control of Velvet Family Regulatory Proteins for Light-Dependent Development and Fungal Cell-Type Specificity
Source: PLoS Genet. 2010 Dec 2;6(12):e1001226. doi: 10.1371/journal.pgen.1001226 (PMC2996326; doi:10.1371/journal.pgen.1001226)
Supplement: Table S1 — Fungal strains used in this study. (0.09 MB DOC) [file pgen.1001226.s007.doc]

**Table S1. Fungal strains used in this study.**

| **Strain** | **Genotype** | **Reference** |
| --- | --- | --- |
| FGSCA4 | *veA+* | FGSC* |
| FGSCA26 | *veA1*, *biA1* | FGSC* |
| TNO2A3 | *nkuA*∆, *pyroA4*, *pyrG89*, *veA1* | [47] |
| AGB152 | *pyroA4*, *pyrG89*, *veA*+ | [48] |
| AGB154 | *pabaA1*, *yA2*, *veA*+ | [49] |
| DVAR1 | *pabaA1*, *yA2*; *argB*∆::*trpC*; *trpC801*; *veA*∆::*argB* | [24] |
| RNI14.1 | *biA1*; *vosA*∆::*argB*^+^; *veA+* | [16] |
| RNI18.3 | *pyroA4*; *velB*∆::*AfpyrG* ^+^, *veA*+ | [16] |
| AGB448 | *niiA-niiD*/*pyrG*, *pyroA4*, *pyrG89*, *veA*+ | [9] |
| AGB389 | *velB::ctap /natR, veA+* | [9] |
| AGB468 | *laeA*∆::*ptrA*; *nkuA*∆, *pyroA4*, *pyrG89*, *veA1* | This study |
| AGB475 | *laeA*-*phleo* ; *laeA*∆::*ptrA*; *nkuA*∆, *pyroA4*, *pyrG89*, *veA1*  (pME3635) | This study |
| AGB493 | *laeA*∆::*ptrA*, *veA*+ | This study |
| AGB494 | *laeA-phleo*; *laeA*∆::*ptrA*, *veA*+ (pME3635) | This study |
| AGB506 | *^p^gpdA::mrfp::h2A / natR; pyroA4*, *pyrG89*, *veA*+ | This study |
| AGB509 | *^p^vosA::vosA::ctap / natR, pyroA4*, *pyrG89*, *veA*+ | This study |
| AGB510 | *^p^vosA::vosA::ctap / natR, veA*+; *laeA*∆::*ptrA* | This study |
| AGB511 | *^p^velB::velB::ctap / natR, veA*+; *laeA*∆::*ptrA* | This study |
| AGB512 | *laeA*∆::*ptrA, veA1* | This study |
| AGB513 | *^p^veA::veA::ctap / natR*; *laeA*∆::*ptrA* (pME3711) | This study |
| AGB514 | *^p^mutA::sgfp, natR*; *pyroA4*, *pyrG89*, *veA*+ (pME3296) | This study |
| AGB515 | *^p^mutA::sgfp, natR*; *laeA*∆::*ptrA* (pME3296) | This study |
| AGB516 | *^p^niiA::n-yfp::velB*/*^p^niiD::c-yfp::vosA*, *pyrG* (BIFC 1, pME3714)  *^p^gpdA::mrfp::h2A / natR; pyroA4*, *pyrG89*, *veA*+ | This study |
| AGB517 | *^p^niiA*::*c-yfp::velB*/*^p^niiD::n-yfp::vosA*, *pyrG* (BIFC 2, pME3715)  *^p^gpdA::mrfp::h2A / natR; pyroA4*, *pyrG89*, *veA*+ | This study |
| AGB518 | *laeA*∆::*ptrA, veA1* (pME3635) | This study |
| AGB519 | *^p^niiA::laeA, phleoR; pyroA4*, *pyrG89*, *veA*+ (pME3716) (OE1) | This study |
| AGB521 | *laeA*∆::*ptrA; veA*∆*::argB* | This study |
| AGB543 | *^p^niiA::n-yfp::velB*/*^p^niiD::c-yfp::velB*, *pyrG; ^p^gpdA::mrfp::h2A / natR; pyroA4*, *pyrG89*, *veA*+ (pME3717) | This study |
| AGB544 | *^p^niiA*::*c-yfp::velB*/*^p^niiD::n-yfp::vosA, laeA*∆::*ptrA*; *nkuA*∆, *pyroA4*, *pyrG89*, *veA1* (BIFC 2, pME3715) | This study |
| AGB545 | *^p^niiA::nosA cDNA, phleoR, laeA*∆*::ptrA, veA+,* | This study |
| AGB546 | *laeA*∆::*ptrA*; *nkuA*∆, *pyroA4*, *pyrG89*, *veA1* (pME3189)  *^p^niiA::n-eyfp::veA / ^p^niiD::c-eyfp::velB, pyrG* | This study |

* Fungal Genetics Stock Center, Kansas City, U.S.A
