# Supplementary material for: LaeA Control of Velvet Family Regulatory Proteins for Light-Dependent Development and Fungal Cell-Type Specificity
Source: PLoS Genet. 2010 Dec 2;6(12):e1001226. doi: 10.1371/journal.pgen.1001226 (PMC2996326; doi:10.1371/journal.pgen.1001226)
Supplement: Table S2 — Plasmids employed in this study. (0.06 MB DOC) [file pgen.1001226.s008.doc]

**Table S2. Plasmids employed in this study.**

| **Plasmid** | **Description** | **Reference** |
| --- | --- | --- |
| pBluescript II SK | Cloning plasmid | Stratagene |
| pPTRII | Pyrithiamine resistance gene (*ptrA*) containing plasmid | Takara |
| pAN8-1 | Phleomycin resistance cloning plasmid | [51] |
| pME3157 | *^p^veA::veA::ctap*, *ptrA*, in pUC19 | [9] |
| pME3160 | *niiA*/*niiD* expression module with *pyrG* marker | [9] |
| pME3189 | *^p^niiA::n-eyfp::veA / ^p^niiD::c-eyfp::velB, pyrG* | [9] |
| pME3296 | *^p^mutA::sgfp, natR* | [49] |
| pME3635 | *laeA* genomic locus in the *Stu*I site of pAN8-1 | This study |
| pME3711 | *^p^veA::veA::ctap*/*natR* | This study |
| pME3712 | *^p^niiA::n-yfp::velB, pyrG* (BIFC recipient 1) | This study |
| pME3713 | *^p^niiA::c-yfp::velB, pyrG* (BIFC recipient 2) | This study |
| pME3714 | *^p^niiA::n-yfp::velB*/*^p^niiD::c-yfp::vosA*, *pyrG* (BIFC 1) | This study |
| pME3715 | *^p^niiA*::*c-yfp::velB*/*^p^niiD::n-yfp::vosA*, *pyrG* (BIFC 2) | This study |
| pME3716 | *^p^niiA::laeA, phleoR* (*laeA* overexpression) | This study |
| pME3717 | *^p^niiA*::*n-yfp::velB*/*^p^niiD::c-yfp::velB*, *pyrG* (BIFC 3) | This study |
| pME3718 | *^p^niiA*-*niiA*^t^ / *^p^niiD*-*niiD*^t^, *phleoR* | This study |
| pME3719 | *^p^niiA*::*nosA, phleoR* | This study |
