# Supplementary material for: LaeA Control of Velvet Family Regulatory Proteins for Light-Dependent Development and Fungal Cell-Type Specificity
Source: PLoS Genet. 2010 Dec 2;6(12):e1001226. doi: 10.1371/journal.pgen.1001226 (PMC2996326; doi:10.1371/journal.pgen.1001226)
Supplement: Table S3 — Oligonucleotides utilized for plasmid constructions and northern hybridizations. (0.09 MB DOC) [file pgen.1001226.s009.doc]

**Table S3. Oligonucleotides utilized for plasmid constructions and northern hybridizations.**

| **Designation** | **Sequence in 5->3 order** | **Features** |
| --- | --- | --- |
| OSB22 | GTA TGG AGT ACA GGA CCG GGT C | *laeA* del A |
| OSB23 | GTA TAA GTT CAG TAG TGT AGT TAG | *laeA* del B *Nested* P |
| OSB24 | CAT TTC GTT ACC AAT GGG ATC CCG TAA TCA ATT GGC GGG GAG ACG AGT TCC C | *laeA* del C  (*ptrA* connector 5) |
| OSB25 | GAA AGA CAG TAT AAT ACA AAC AAA GAT GCA AGA GAG CAA AAG GCG ACC ACA TCC | *laeA* del D  (*ptrA* connector 3) |
| OSB26 | CAG AAA TGG TCG GCA CTC GC | *laeA* del E *Nested* P |
| OSB27 | CGT TGT AGA ATG GCA TCC AAC C | *laeA* del F |
| OSB28 | GTA TAG ATC AGC GGC ACC CGA TTC | *ptrA* 3 <-5 binder |
| OZG29 | CTA CTT GTA CAG TTC GTC CAT GCC GTG | *gfp* stop |
| OZG64 | TTA GTA TTC GTT ATC CAG ACC ATC G | VelB-3 |
| OZG73 | ATG GTG AGC AAG GGC GAG GAG | *n-yfp* start |
| OZG75 | ATG GCC GAC AAG CAG AAG AAC | *c-yfp* start |
| OZG77 | GGA ATG CGC CCT ATC CTC AAC AGC GTA CAT GTG GTT CAT GAC CTT CTG TTT CAG | VelB-CYFP |
| OZG167 | TCA GGT TGA CTT CCC CGC GGA ATT CG | *ctap* stop |
| VosA-A | CGA AAT ACA CGG TCG GGG TTA CTC | 5 UTR amplifier |
| VosA-B | GCA TTA AAG GCA GAT ACG AGA TAG | 5 UTR *nested* Primer |
| VosA-C | GAA ATT CTT TTT CCA TCT TCT CTT ACC ACC GCT ACC ACC CCG AGG AGT TCC GTT CGC TGAG | VosA-C TAP connector |
| VosA-D | GAG CAG GCG CTC TAC ATG AGC ATG CCC TGC CCC TGA GGA TTC TCG TTT GTG GAA CAC CTG | *natR*- 3 UTR connector |
| VosA-E | CCT TGA GAA CTC CAT GCG TGT CG | 3 UTR *nested* Primer |
| VosA-F | GAT CCG CTG GAC TTG CTG GTG | 3 UTR amplifier |
| OZG304 | TTT TGG GCC CAA GCT TGG TGA TCC TCG TCT TCG G | VeA 5 *Apa*I |
| OZG305 | TTT TGG GCC CAA GCT TAT CCT CCA GGT TAC TGA CTC | VeA 3 *Apa*I |
| OZG387 | CGT GGC GAT GGA GCG CAT GAT ATA | NYFP 3 |
| OZG388 | GTG GTT CAT GAC CTT CTG TTT CAG GTC | CYFP3 |
| OZG397 | CAA CTA CAA CAG CCA CAA CGT CTA TAT CAT GCG CTC CAT CGC CAC GAT GTA CGC TG | VelB-NYFP |
| OZG436 | CAA CGT CTA TAT CAT GCG CTC CAT CGC CAC GAT GAG TGC GGC GAA CTA TCC AG | VosA-NYFP |
| OZG437 | GAA CGA CCT GAA ACA GAA GGT CAT GAA CCA CAT GAG TGC GGC GAA CTA TCC AG | VosA-CYFP |
| OZG438 | TTT AAT CAC CGA GGA GTT CCG TTC GCT G | VosA 3 |
| OMN176 | CCA TCA CCA TAA AGC GAT CAG | *tpsA* northern |
| OMN177 | CAG TTT CGA GAA GTT AAG CGC | *tpsA* northern |
| OMN182 | CAG CCG CAT CTC CAA CTT AG | *orlA* northern |
| OMN183 | TGT TAG CAG CAA TTC ATC GCG | *orlA* northern |
| *brlA 5* | ATG CGA AAT CAG TCC AGC CTG TCC G | *brlA* northern |
| *brlA 3* | TCA TTC ATC CCA GCC GTC CAG GCT C | *brlA* northern |
| *abaA 5* | ATG GCT ACT GAC TGG CAA CCC GAG | *abaA* northern |
| *abaA 3* | CTA GAC AGC CTC AAC CGC AGT ATG | *abaA* northern |
| *mutA 5* | ATG AAG ATC TTC CAC CGC TGC TG | *mutA* northern |
| *mutA 3* | TAG GCG CTA AAA GAG CCA ACA T | *mutA* northern |
| *nosA 5*  (OZG320) | ATG CCG GCA GCA CCG AGA AAG AAG | *nosA* northern |
| *nosA 3*  (OZG321) | TCA AAG AAG AAG GTA GTT CCA ACC G | *nosA* northern |
| *steA 5* | TTA TGT ACT CTC AGC ACG GTG CCC C | *steA* northern |
| *steA 3* | TTC TAT ATT TGC TGT TGC AGG AGT TG | *steA* northern |
| *nsdD 5* | ATG GGA TCA CTA GAG GCT GGA CAT AG | *nsdD* northern |
| *nsdD 3* | TTA ATG ACT CCT CGG TGA CAC CG | *nsdD* northern |
| *laeA 5* | GAA TTC ATG TTT GAG ATG GGC CCG GTG GG | *laeA* northern |
| *laeA 3* | CTC GAG TTA TCT TAA TGG TTT CCT AGC CTG GT | *laeA* northern |
| *aflR 5* | ATG GAG CCC CCA GCG ATC AGC CAG | *aflR* northern |
| *aflR 3* | TCA GGC GTG GCG GAG GAT GCT GAT C | *aflR* northern |
| *ipnA 5* | ATG GGT TCA GTC AGC AAA GCC AAT G | *ipnA* northern |
| *ipnA 3* | CTA GGT CTG GCC GTT CTT GTT G | *ipnA* northern |
| *stcU5* | ATG TCC TCC TCC GAT AAT TAC CG | *stcU* northern |
| *stcU3* | TTA TCT AAA GGC CCC CCC ATC AAC G | *stcU* northern |
| *gpdA 5* | ATG GCA CCA ACA AAG AAA CAC CAG | *gpdA* northern |
| *gpdA 3* | CTA TTG GGC ATC AAC CTT GGA G | *gpdA* northern |
