# Supplementary material for: LaeA Control of Velvet Family Regulatory Proteins for Light-Dependent Development and Fungal Cell-Type Specificity
Source: PLoS Genet. 2010 Dec 2;6(12):e1001226. doi: 10.1371/journal.pgen.1001226 (PMC2996326; doi:10.1371/journal.pgen.1001226)
Supplement: Table S4 — SEQUEST Multiple Consensus Report of VosA::cTAP tag identifications after nano-LC-ESI-MS2. (0.06 MB DOC) [file pgen.1001226.s010.doc]

**Table S4.** **SEQUEST Multiple Consensus Report of VosA::cTAP tag identifications after nano-LC-ESI-MS2.**

**Light and dark cultures**

| **Reference (database entry) -- Average Mass -- pI -- Coverage (amino acids)** | | | | | | **Score** |  | **Peptides** |
| --- | --- | --- | --- | --- | --- | --- | --- | --- |
| **File, Scan(s)** | **Sequence** | **MH+** | **Charge** | **XCorr** | **Delta Cn** | **Sp** | **RSp** | **Ions** |
| **AN1959 (VosA) -- 48980.2 -- 8.8 -- 40.7%** | | | | | | **158.2** |  | **16 (15-1-0-0-0)** |
| 712, OB18_dark | R.PQYSASTAVLPPLQQSR.N | 1842.97 | 2 | 4.36 | 0.57 | 851.1 | 1 | 23/32 |
| 648, OB18_dark | R.TAM*QIPGSSYPAPPYQPTSR.D | 2165.03 | 2 | 3.59 | 0.46 | 625.8 | 1 | 20/38 |
| 724, OB17_dark | R.TAMQIPGSSYPAPPYQPTSR.D | 2149.03 | 2 | 4.50 | 0.56 | 987.6 | 1 | 24/38 |
| 815, OB16_dark | R.PSTSDDFELIVR.Q | 1378.68 | 2 | 3.73 | 0.54 | 1185.0 | 1 | 16/22 |
| 858, OB17_dark | K.SFPGMAESTFLSR.S | 1429.67 | 2 | 2.51 | 0.49 | 380.4 | 1 | 16/24 |
| 718, OB16_dark | R.SSQQATMQSLGMVNPPGTPTPDSAR.A | 2558.19 | 2 | 4.36 | 0.61 | 862.0 | 1 | 23/48 |
| 544, OB16_dark | R.TAPRPEEYPQAAIPR.S | 1695.88 | 3 | 3.45 | 0.46 | 1266.1 | 1 | 26/56 |
| 393, OB16_dark | R.AMM*QQAYPR.P | 1111.50 | 2 | 2.84 | 0.03 | 983.3 | 1 | 13/16 |
| 774, OB17_dark | R.GYYEQSPQATPILPSQPLGTSEAER.Y | 2719.32 | 2 | 2.86 | 0.56 | 445.8 | 1 | 18/48 |
| 575, OB18_dark | R.SSQQATM*QSLGM*VNPPGTPTPDSAR.A | 2590.19 | 2 | 3.70 | 0.59 | 454.2 | 1 | 18/48 |
| 414, OB16_dark | R.YGVPPGHTGYDHTGSANGTPR.- | 2140.97 | 2 | 2.19 | 0.54 | 395.1 | 1 | 15/40 |
| 502, OB16_dark | R.AMMQQAYPR.P | 1095.50 | 2 | 3.21 | 0.39 | 1209.5 | 1 | 14/16 |
| 628, OB16_dark | R.DYSYYAPVK.R | 1105.52 | 1 | 1.99 | 0.43 | 489.3 | 1 | 10/16 |
| 787, OB16_dark | K.FSLFEM*R.K | 945.45 | 2 | 1.92 | 0.08 | 717.5 | 1 | 11/12 |
| 395, OB17_dark | R.AM*MQQAYPR.P | 1111.50 | 2 | 2.07 | 0.31 | 543.6 | 2 | 11/16 |
| 415, OB16_dark | R.SFADQGVK.L | 851.42 | 1 | 1.65 | 0.27 | 467.9 | 1 | 9/14 |
| **AN0363 (VelB) -- 40015.7 -- 6.1 -- 23.9%** | | | | | | **80.3** |  | **8 (8-0-0-0-0)** |
| 794, OB18_dark | K.SVSDLPQSDIAEVINK.G | 1714.88 | 2 | 5.00 | 0.62 | 909.0 | 1 | 23/30 |
| 857, OB17_dark | R.IWSLQVVQQPIR.A | 1466.84 | 2 | 4.21 | 0.43 | 1855.8 | 1 | 17/22 |
| 662, OB18_dark | K.KFPGVIESTPLSK.V | 1402.79 | 2 | 3.34 | 0.41 | 771.9 | 1 | 18/24 |
| 792, OB17_dark | R.NLIGCLSASAYR.L | 1324.66 | 2 | 3.60 | 0.53 | 1630.6 | 1 | 19/22 |
| 399, OB17_dark | R.MCGFGDKDR.R | 1085.45 | 2 | 3.08 | 0.46 | 751.7 | 1 | 12/16 |
| 477, OB17_dark | R.RPITPPPCIR.L | 1206.67 | 2 | 1.67 | 0.36 | 270.7 | 1 | 13/18 |
| 452, OB17_dark | K.VFANQGIK.I | 876.49 | 1 | 1.52 | 0.22 | 347.7 | 1 | 10/14 |
| 729, OB17_dark | K.FSFVNVGK.S | 897.48 | 2 | 2.36 | 0.11 | 601.8 | 1 | 12/14 |

Neither AN1959 (VosA) nor AN0363 (VelB) could be identified from samples of cultures grown in the light.
