# Supplementary material for: LaeA Control of Velvet Family Regulatory Proteins for Light-Dependent Development and Fungal Cell-Type Specificity
Source: PLoS Genet. 2010 Dec 2;6(12):e1001226. doi: 10.1371/journal.pgen.1001226 (PMC2996326; doi:10.1371/journal.pgen.1001226)
Supplement: Table S5 — SEQUEST Multiple Consensus Report of VosA::cTAP tag identifications in laeAΔ after nano-LC-ESI-MS2. (0.09 MB DOC) [file pgen.1001226.s011.doc]

**Table S5.** **SEQUEST Multiple Consensus Report of VosA::cTAP tag identifications in *laeA***∆ **after nano-LC-ESI-MS2.**

A) **Light cultures**

| **Reference (database entry) -- Average Mass -- pI -- Coverage (amino acids)** | | | | | | **Score** |  | **Peptides** |
| --- | --- | --- | --- | --- | --- | --- | --- | --- |
| **File, Scan(s)** | **Sequence** | **MH+** | **Charge** | **XCorr** | **Delta Cn** | **Sp** | **RSp** | **Ions** |
| **AN1959 (VosA) -- 48980.2 -- 8.8 -- 45.2%** | | | | | | **198.2** |  | **20 (19-1-0-0-0)** |
| 707, OB19_light | R.PQYSASTAVLPPLQQSR.N | 1842.97 | 2 | 4.33 | 0.57 | 1110.3 | 1 | 25/32 |
| 643, OB20_light | R.TAM*QIPGSSYPAPPYQPTSR.D | 2165.03 | 2 | 4.21 | 0.59 | 809.0 | 1 | 21/38 |
| 572, OB19_light | R.SSQQATM*QSLGM*VNPPGTPTPDSAR.A | 2590.19 | 2 | 4.41 | 0.68 | 590.6 | 1 | 20/48 |
| 812, OB19_light | R.PSTSDDFELIVR.Q | 1378.68 | 2 | 4.08 | 0.53 | 1626.5 | 1 | 18/22 |
| 564, OB20_light | R.TAPRPEEYPQAAIPR.S | 1695.88 | 2 | 3.69 | 0.55 | 656.3 | 1 | 15/28 |
| 719, OB19_light | R.SSQQATMQSLGMVNPPGTPTPDSAR.A | 2558.19 | 2 | 3.97 | 0.61 | 710.4 | 1 | 22/48 |
| 653, OB19_light | R.SSQQATMQSLGM*VNPPGTPTPDSAR.A | 2574.19 | 2 | 3.93 | 0.09 | 559.8 | 1 | 21/48 |
| 723, OB19_light | R.TAMQIPGSSYPAPPYQPTSR.D | 2149.03 | 2 | 4.12 | 0.58 | 706.5 | 1 | 20/38 |
| 649, OB20_light | R.DYSYYAPVK.R | 1105.52 | 1 | 2.18 | 0.23 | 480.1 | 1 | 10/16 |
| 419, OB19_light | R.YGVPPGHTGYDHTGSANGTPR.- | 2140.97 | 3 | 1.85 | 0.19 | 284.1 | 1 | 24/80 |
| 773, OB20_light | K.SFPGM*AESTFLSR.S | 1445.67 | 2 | 2.36 | 0.51 | 297.1 | 1 | 15/24 |
| 764, OB19_light | R.GYYEQSPQATPILPSQPLGTSEAER.Y | 2719.32 | 2 | 3.25 | 0.61 | 306.4 | 1 | 15/48 |
| 653, OB19_light | R.SSQQATM*QSLGMVNPPGTPTPDSAR.A | 2574.19 | 2 | 3.60 | 0.67 | 487.8 | 2 | 20/48 |
| 512, OB20_light | R.AMMQQAYPR.P | 1095.50 | 2 | 2.65 | 0.36 | 950.4 | 1 | 13/16 |
| 549, OB20_light | R.DYSYYAPVKR.Q | 1261.62 | 2 | 1.98 | 0.37 | 386.1 | 1 | 9/18 |
| 902, OB19_light | K.FSLFEMR.K | 929.45 | 2 | 2.32 | 0.40 | 635.8 | 1 | 10/12 |
| 417, OB20_light | R.SFADQGVK.L | 851.42 | 1 | 1.35 | 0.38 | 195.9 | 1 | 7/14 |
| 517, OB19_light | R.FTVSPPK.S | 775.43 | 1 | 1.46 | 0.16 | 206.0 | 2 | 7/12 |
| 798, OB20_light | K.FSLFEM*R.K | 945.45 | 2 | 1.95 | 0.23 | 800.3 | 2 | 10/12 |
| 618, OB21_light | R.KPVDPPPIVQIR.V | 1358.81 | 2 | 1.80 | 0.50 | 286.8 | 1 | 13/22 |
| **AN0363 (VelB) -- 40015.7 -- 6.1 -- 23.9%** | | | | | | **80.2** |  | **8 (8-0-0-0-0)** |
| 797, OB19_light | K.SVSDLPQSDIAEVINK.G | 1714.88 | 2 | 3.94 | 0.50 | 842.8 | 1 | 21/30 |
| 658, OB20_light | K.KFPGVIESTPLSK.V | 1402.79 | 2 | 3.49 | 0.47 | 896.9 | 1 | 20/24 |
| 869, OB20_light | R.IWSLQVVQQPIR.A | 1466.84 | 2 | 4.31 | 0.42 | 1647.0 | 1 | 17/22 |
| 813, OB20_light | R.NLIGCLSASAYR.L | 1324.66 | 2 | 3.28 | 0.48 | 1190.7 | 1 | 17/22 |
| 409, OB20_light | R.MCGFGDKDR.R | 1085.45 | 2 | 3.08 | 0.41 | 1087.8 | 1 | 14/16 |
| 739, OB20_light | K.FSFVNVGK.S | 897.48 | 2 | 2.11 | 0.26 | 618.2 | 1 | 12/14 |
| 462, OB20_light | K.VFANQGIK.I | 876.49 | 1 | 1.64 | 0.23 | 501.2 | 1 | 11/14 |
| 482, OB20_light | R.RPITPPPCIR.L | 1206.67 | 2 | 2.13 | 0.31 | 260.2 | 1 | 12/18 |

B) **Dark cultures**

| **Reference (database entry) -- Average Mass -- pI -- Coverage (amino acids)** | | | | | | **Score** |  | **Peptides** |
| --- | --- | --- | --- | --- | --- | --- | --- | --- |
| **File, Scan(s)** | **Sequence** | **MH+** | **Charge** | **XCorr** | **Delta Cn** | **Sp** | **RSp** | **Ions** |
| **AN1959 (VosA) -- 48980.2 -- 8.8 -- 16.4%** | | | | | | **50.2** |  | **5 (5-0-0-0-0)** |
| 730, OB23_dark | R.PQYSASTAVLPPLQQSR.N | 1842.97 | 2 | 3.01 | 0.51 | 663.4 | 1 | 20/32 |
| 663, OB24_dark | R.TAM*QIPGSSYPAPPYQPTSR.D | 2165.03 | 2 | 3.33 | 0.60 | 575.9 | 1 | 19/38 |
| 569, OB23_dark | R.TAPRPEEYPQAAIPR.S | 1695.88 | 2 | 4.09 | 0.42 | 1106.2 | 1 | 19/28 |
| 694, OB22_dark | R.DYSYYAPVK.R | 1105.52 | 2 | 2.11 | 0.45 | 404.2 | 1 | 10/16 |
| 619, OB23_dark | R.KPVDPPPIVQIR.V | 1358.81 | 2 | 2.02 | 0.34 | 217.0 | 1 | 10/22 |
| **AN0363 (VelB) -- 40015.7 -- 6.1 -- 10.8%** | | | | | | **30.2** |  | **3 (3-0-0-0-0)** |
| 824, OB24_dark | K.SVSDLPQSDIAEVINK.G | 1714.88 | 2 | 4.27 | 0.59 | 669.5 | 1 | 20/30 |
| 868, OB23_dark | R.IWSLQVVQQPIR.A | 1466.84 | 2 | 3.93 | 0.44 | 1658.6 | 1 | 16/22 |
| 813, OB24_dark | R.NLIGCLSASAYR.L | 1324.66 | 2 | 2.88 | 0.55 | 979.6 | 1 | 16/22 |
