# Supplementary material for: LaeA Control of Velvet Family Regulatory Proteins for Light-Dependent Development and Fungal Cell-Type Specificity
Source: PLoS Genet. 2010 Dec 2;6(12):e1001226. doi: 10.1371/journal.pgen.1001226 (PMC2996326; doi:10.1371/journal.pgen.1001226)
Supplement: Table S6 — SEQUEST Multiple Consensus Report of VeA::cTAP tag identifications in laeAΔ after nano-LC-ESI-MS2. (0.19 MB DOC) [file pgen.1001226.s012.doc]

**Table S6.** **SEQUEST Multiple Consensus Report of VeA::cTAP tag identifications in *laeA***∆ **after nano-LC-ESI-MS2.**

A) **Dark cultures**

| **Reference (database entry) -- Average Mass -- pI -- Coverage (amino acids)** | | | | | | **Score** |  | **Peptides** |
| --- | --- | --- | --- | --- | --- | --- | --- | --- |
| **File, Scan(s)** | **Sequence** | **MH+** | **Charge** | **XCorr** | **Delta Cn** | **Sp** | **RSp** | **Ions** |
| **AN1052 (VeA) -- 59282.3 -- 9.5 -- 42.5%** | | | | | | **240.3** |  | **24 (24-0-0-0-0)** |
| 687, OB135_dark | R.NQSISEYEPSMGYPGSQTR.L | 2130.94 | 2 | 4.65 | 0.62 | 906.9 | 1 | 22/36 |
| 615, OB135_dark | R.NQSISEYEPSM*GYPGSQTR.L | 2146.94 | 2 | 4.05 | 0.63 | 559.6 | 1 | 18/36 |
| 542, OB135_dark | R.RPSAVEYGQPIAQPYQR.P | 1960.00 | 2 | 4.61 | 0.61 | 711.4 | 1 | 20/32 |
| 652, OB135_dark | R.PSYGQPSQTTSLPPLR.H | 1728.89 | 2 | 4.40 | 0.68 | 1096.1 | 1 | 23/30 |
| 672, OB137_dark | R.STSISTNMDPYSYPSR.R | 1805.80 | 2 | 4.33 | 0.73 | 1416.4 | 1 | 20/30 |
| 577, OB135_dark | R.STSISTNM*DPYSYPSR.R | 1821.80 | 2 | 3.70 | 0.68 | 1035.6 | 1 | 17/30 |
| 494, OB135_dark | R.RPDQYAGSDAYANAPERPR.S | 2134.01 | 3 | 4.90 | 0.53 | 1078.4 | 1 | 29/72 |
| 618, OB137_dark | R.LSAERPSYGQPSQTTSLPPLR.H | 2285.19 | 2 | 3.73 | 0.68 | 593.2 | 1 | 19/40 |
| 907, OB136_dark | R.LEVISNPFIVYSAK.K | 1579.87 | 2 | 4.15 | 0.58 | 1701.1 | 1 | 23/26 |
| 907, OB135_dark | K.LSSPQEFLEFR.L | 1352.68 | 2 | 2.88 | 0.52 | 724.5 | 1 | 14/20 |
| 460, OB135_dark | R.RPDQYAGSDAYANAPER.P | 1880.85 | 2 | 4.29 | 0.66 | 1025.4 | 1 | 20/32 |
| 702, OB139_dark | R.SSLLDGPDQMAYK.R | 1424.67 | 2 | 3.52 | 0.43 | 1141.1 | 1 | 18/24 |
| 399, OB135_dark | K.RTEDYDYDNER.G | 1475.60 | 2 | 3.19 | 0.42 | 1204.7 | 1 | 16/20 |
| 602, OB138_dark | R.SSLLDGPDQM*AYK.R | 1440.67 | 2 | 3.77 | 0.42 | 933.4 | 1 | 17/24 |
| 997, OB135_dark | R.AGYFIFPDLSVR.N | 1384.73 | 2 | 3.59 | 0.50 | 988.8 | 1 | 17/22 |
| 688, OB139_dark | R.LWETNSMLSK.R | 1208.60 | 2 | 3.19 | 0.32 | 661.1 | 1 | 15/18 |
| 594, OB135_dark | R.LWETNSM*LSK.R | 1224.60 | 2 | 2.59 | 0.51 | 630.7 | 1 | 15/18 |
| 437, OB136_dark | R.TEDYDYDNER.G | 1319.50 | 2 | 3.71 | 0.56 | 911.6 | 1 | 15/18 |
| 709, OB135_dark | K.KFPGLTTSTPISR.M | 1404.78 | 2 | 2.81 | 0.46 | 579.5 | 1 | 15/24 |
| 362, OB136_dark | R.HSLEPSVNSR.S | 1125.56 | 2 | 2.78 | 0.31 | 508.1 | 1 | 13/18 |
| 550, OB136_dark | K.DATEGTQPMPSPVPGK.L | 1611.77 | 2 | 3.26 | 0.57 | 686.1 | 1 | 18/30 |
| 798, OB135_dark | K.LM*TNQGSPVLTGVPVAGVAYLDKPNR.A | 2713.44 | 3 | 4.92 | 0.66 | 1126.0 | 1 | 33/100 |
| 449, OB135_dark | K.DATEGTQPM*PSPVPGK.L | 1627.77 | 2 | 3.36 | 0.57 | 718.8 | 1 | 19/30 |
| 699, OB136_dark | K.FPGLTTSTPISR.M | 1276.69 | 2 | 2.88 | 0.42 | 549.5 | 1 | 13/22 |

| **Reference (database entry) -- Average Mass -- pI -- Coverage (amino acids)** | | | | | | | **Score** |  | **Peptides** |
| --- | --- | --- | --- | --- | --- | --- | --- | --- | --- |
| **File, Scan(s)** | **Sequence** | **MH+** | **Charge** | | **XCorr** | **Delta Cn** | **Sp** | **RSp** | **Ions** |
| **AN0363 (VelB) -- 40015.7 -- 6.1 -- 33.6%** | | | | | | | **140.3** |  | **14 (14-0-0-0-0)** |
| 903, OB137_dark | K.SVSDLPQSDIAEVINK.G | 1714.89 | | 2 | 3.61 | 0.52 | 792.5 | 1 | 21/30 |
| 782, OB137_dark | R.NLIGCLSASAYR.L | 1324.67 | | 2 | 4.04 | 0.56 | 1353.3 | 1 | 18/22 |
| 807, OB138_dark | R.IWSLQVVQQPIR.A | 1466.85 | | 2 | 3.88 | 0.40 | 2053.7 | 1 | 18/22 |
| 474, OB137_dark | R.GRHSDEDDGLDNEY.- | 1621.64 | | 2 | 2.74 | 0.58 | 849.1 | 1 | 16/26 |
| 627, OB139_dark | K.KFPGVIESTPLSK.V | 1402.79 | | 2 | 3.66 | 0.53 | 1307.0 | 1 | 20/24 |
| 1017, OB137_dark | K.GTAPILASTFSEPFQVFSAK.K | 2098.09 | | 2 | 4.27 | 0.51 | 919.4 | 1 | 21/38 |
| 737, OB137_dark | K.FPGVIESTPLSK.V | 1274.70 | | 2 | 3.30 | 0.47 | 1166.8 | 1 | 19/22 |
| 688, OB138_dark | K.FSFVNVGK.S | 897.48 | | 2 | 2.20 | 0.34 | 707.7 | 1 | 13/14 |
| 428, OB139_dark | K.VFANQGIK.I | 876.49 | | 1 | 1.50 | 0.18 | 295.9 | 1 | 9/14 |
| 564, OB137_dark | R.HSDEDDGLDNEY.- | 1408.51 | | 2 | 2.48 | 0.49 | 628.6 | 1 | 14/22 |
| 402, OB137_dark | R.MCGFGDKDR.R | 1085.45 | | 2 | 2.88 | 0.46 | 1074.6 | 1 | 14/16 |
| 463, OB137_dark | R.RPITPPPCIR.L | 1206.68 | | 2 | 2.76 | 0.36 | 540.4 | 1 | 14/18 |
| 835, OB137_dark | R.LKFSFVNVGK.S | 1138.66 | | 2 | 1.77 | 0.39 | 274.2 | 3 | 10/18 |
| 424, OB137_dark | R.M*CGFGDKDR.R | 1101.45 | | 2 | 2.04 | 0.17 | 607.2 | 1 | 12/16 |

| **AN02142 (KapA) -- 60589.8 -- 4.9 -- 42.5%** | | | | | | **214.3** |  | **22 (22-0-0-0-0)** |
| --- | --- | --- | --- | --- | --- | --- | --- | --- |
| 345, OB139 | K.VGEMDKEAGQGDAHVNR.Y | 1812.83 | 3 | 4.74 | 0.63 | 3274.1 | 1 | 35/64 |
| 468, OB136 | K.IHDCQNNANEEIYMK.A | 1878.81 | 2 | 5.13 | 0.66 | 2514.5 | 1 | 23/28 |
| 843, OB135 | R.EQAVWALGNIAGDSPQCR.D | 1971.93 | 2 | 5.39 | 0.69 | 1804.2 | 1 | 23/34 |
| 824, OB138 | K.GVFSDQIEAQIQATTK.F | 1735.89 | 2 | 5.18 | 0.63 | 1626.5 | 1 | 21/30 |
| 685, OB135 | K.EACWAISNATSGGLQKPDQIR.Y | 2302.12 | 3 | 3.84 | 0.55 | 904.9 | 1 | 31/80 |
| 842, OB135 | R.YALFIEEAGGMEK.I | 1457.70 | 2 | 4.34 | 0.57 | 2020.0 | 1 | 22/24 |
| 1029, OB137 | K.IIQVALDGLENILK.V | 1538.92 | 2 | 4.71 | 0.57 | 2244.1 | 1 | 21/26 |
| 332, OB137 | R.RREEQQVEIR.K | 1342.72 | 3 | 2.99 | 0.25 | 1025.2 | 1 | 24/36 |
| 683, OB135 | K.IQAVIEAGIPR.R | 1166.69 | 2 | 4.52 | 0.41 | 2372.9 | 1 | 19/20 |
| 768, OB136 | R.NATWTLSNFCR.G | 1369.63 | 2 | 3.28 | 0.58 | 883.1 | 1 | 14/20 |
| 302, OB136 | K.VGEM*DKEAGQGDAHVNR.Y | 1828.83 | 3 | 4.69 | 0.59 | 2741.4 | 1 | 35/64 |
| 469, OB137 | R.VIETGVVSR.F | 959.55 | 2 | 3.13 | 0.45 | 886.1 | 1 | 15/16 |
| 947, OB135 | K.TPQPDWNTIAPALPVLAK.L | 1932.06 | 2 | 3.88 | 0.63 | 943.1 | 1 | 18/34 |

| 358, OB135_dark | R.REEQQVEIR.K | 1186.62 | 2 | 3.62 | 0.22 | 1179.5 | 1 | 15/16 |
| --- | --- | --- | --- | --- | --- | --- | --- | --- |
| 793, OB136_dark | R.PLLTLINDGR.K | 1111.65 | 2 | 3.27 | 0.36 | 1518.0 | 1 | 15/18 |
| 307, OB135_dark | K.ERNPPIER.V | 1010.54 | 2 | 2.97 | 0.32 | 892.9 | 2 | 12/14 |
| 413, OB137_dark | K.IHDCQNNANEEIYM*K.A | 1894.81 | 3 | 3.20 | 0.35 | 625.8 | 1 | 24/56 |
| 532, OB135_dark | K.AYNIIEK.Y | 850.47 | 2 | 2.64 | 0.31 | 571.4 | 2 | 11/12 |
| 504, OB135_dark | R.NQFRPDELR.R | 1174.60 | 2 | 2.34 | 0.09 | 773.6 | 2 | 13/16 |
| 413, OB136_dark | R.EEQQVEIR.K | 1030.52 | 2 | 2.14 | 0.17 | 409.0 | 5 | 10/14 |
| 444, OB135_dark | R.NQFRPDELRR.R | 1330.70 | 3 | 2.37 | 0.15 | 530.2 | 4 | 16/36 |
| 724, OB136_dark | R.FVEFLR.S | 810.45 | 1 | 1.88 | 0.16 | 321.4 | 3 | 8/10 |

B) **Light cultures**

| **Reference (database entry) -- Average Mass -- pI -- Coverage (amino acids)** | | | | | | **Score** |  | **Peptides** |
| --- | --- | --- | --- | --- | --- | --- | --- | --- |
| **File, Scan(s)** | **Sequence** | **MH+** | **Charge** | **XCorr** | **Delta Cn** | **Sp** | **RSp** | **Ions** |
| **AN1052 (VeA) -- 59282.3 -- 9.5 -- 39.3%** | | | | | | **240.3** |  | **24 (24-0-0-0-0)** |
| 662, OB134_light | R.NQSISEYEPSMGYPGSQTR.L | 2130.94 | 2 | 5.02 | 0.71 | 910.5 | 1 | 22/36 |
| 432, OB130_light | R.RPDQYAGSDAYANAPERPR.S | 2134.01 | 3 | 5.91 | 0.62 | 2138.6 | 1 | 39/72 |
| 982, OB132_light | R.AGYFIFPDLSVR.N | 1384.73 | 2 | 3.62 | 0.49 | 961.7 | 1 | 17/22 |
| 644, OB130_light | R.PSYGQPSQTTSLPPLR.H | 1728.89 | 2 | 4.24 | 0.60 | 849.9 | 1 | 20/30 |
| 567, OB131_light | R.STSISTNM*DPYSYPSR.R | 1821.80 | 2 | 3.87 | 0.67 | 1372.6 | 1 | 18/30 |
| 658, OB132_light | R.STSISTNMDPYSYPSR.R | 1805.80 | 2 | 3.12 | 0.60 | 568.0 | 1 | 14/30 |
| 922, OB130_light | R.LEVISNPFIVYSAK.K | 1579.87 | 2 | 4.32 | 0.56 | 1182.2 | 1 | 19/26 |
| 592, OB134_light | R.NQSISEYEPSM*GYPGSQTR.L | 2146.94 | 2 | 3.58 | 0.70 | 781.3 | 1 | 21/36 |
| 617, OB131_light | R.SSLLDGPDQM*AYK.R | 1440.67 | 2 | 3.46 | 0.41 | 1081.4 | 1 | 19/24 |
| 438, OB131_light | R.TEDYDYDNER.G | 1319.50 | 2 | 2.96 | 0.55 | 689.8 | 1 | 13/18 |
| 530, OB132_light | R.RPSAVEYGQPIAQPYQR.P | 1960.00 | 3 | 3.94 | 0.42 | 1177.0 | 1 | 28/64 |
| 388, OB130_light | K.RTEDYDYDNER.G | 1475.60 | 2 | 3.38 | 0.49 | 1147.6 | 1 | 15/20 |
| 707, OB131_light | R.SSLLDGPDQMAYK.R | 1424.67 | 2 | 3.15 | 0.25 | 898.0 | 1 | 17/24 |
| 692, OB134_light | R.LWETNSMLSK.R | 1208.60 | 2 | 2.81 | 0.30 | 678.0 | 1 | 15/18 |
| 618, OB130_light | R.LSAERPSYGQPSQTTSLPPLR.H | 2285.19 | 2 | 3.11 | 0.51 | 499.6 | 1 | 16/40 |
| 624, OB130_light | R.PSAVEYGQPIAQPYQR.P | 1803.90 | 2 | 4.36 | 0.54 | 906.5 | 1 | 18/30 |
| 856, OB130_light | K.LSSPQEFLEFR.L | 1352.68 | 2 | 3.05 | 0.51 | 697.4 | 1 | 15/20 |
| 598, OB131_light | K.KFPGLTTSTPISR.M | 1404.78 | 2 | 2.82 | 0.39 | 593.5 | 1 | 15/24 |
| 561, OB130_light | K.DATEGTQPMPSPVPGK.L | 1611.77 | 2 | 4.00 | 0.60 | 801.1 | 1 | 19/30 |
| 708, OB131_light | K.FPGLTTSTPISR.M | 1276.69 | 2 | 3.07 | 0.45 | 859.6 | 1 | 16/22 |
| 356, OB130_light | R.HSLEPSVNSR.S | 1125.56 | 2 | 2.45 | 0.35 | 355.0 | 2 | 11/18 |
| 451, OB130_light | K.DATEGTQPM*PSPVPGK.L | 1627.77 | 2 | 2.87 | 0.64 | 584.9 | 1 | 18/30 |
| 329, OB130_light | R.MVSKPATMR.- | 1020.53 | 3 | 2.35 | 0.29 | 353.3 | 1 | 13/32 |

| **Reference (database entry) -- Average Mass -- pI -- Coverage (amino acids)** | | | | | | | **Score** |  | **Peptides** |
| --- | --- | --- | --- | --- | --- | --- | --- | --- | --- |
| **File, Scan(s)** | **Sequence** | **MH+** | **Charge** | | **XCorr** | **Delta Cn** | **Sp** | **RSp** | **Ions** |
| **AN0363 (VelB) -- 40015.7 -- 6.1 -- 34.2%** | | | | | | | **120.3** |  | **12 (12-0-0-0-0)** |
| 814, OB132_light | K.SVSDLPQSDIAEVINK.G | 1714.89 | | 2 | 4.03 | 0.59 | 788.1 | 1 | 21/30 |
| 837, OB131_light | R.IWSLQVVQQPIR.A | 1466.85 | | 2 | 4.18 | 0.41 | 2233.8 | 1 | 18/22 |
| 1002, OB132_light | K.GTAPILASTFSEPFQVFSAK.K | 2098.09 | | 2 | 4.62 | 0.47 | 936.8 | 1 | 21/38 |
| 732, OB133_light | R.NLIGCLSASAYR.L | 1324.67 | | 2 | 3.56 | 0.51 | 1454.5 | 1 | 19/22 |
| 648, OB131_light | K.KFPGVIESTPLSK.V | 1402.79 | | 2 | 2.67 | 0.34 | 491.9 | 1 | 16/24 |
| 712, OB132_light | K.FPGVIESTPLSK.V | 1274.70 | | 2 | 3.01 | 0.49 | 979.4 | 1 | 18/22 |
| 390, OB132_light | R.MCGFGDKDR.R | 1085.45 | | 2 | 2.78 | 0.41 | 1155.1 | 1 | 14/16 |
| 713, OB131_light | K.FSFVNVGK.S | 897.48 | | 2 | 2.43 | 0.23 | 596.1 | 1 | 12/14 |
| 560, OB132_light | R.HSDEDDGLDNEY.- | 1408.51 | | 2 | 2.16 | 0.47 | 407.0 | 1 | 11/22 |
| 433, OB132_light | K.VFANQGIK.I | 876.49 | | 1 | 1.80 | 0.27 | 382.8 | 1 | 10/14 |
| 442, OB132_light | R.RPITPPPCIR.L | 1206.68 | | 2 | 2.85 | 0.44 | 386.7 | 1 | 12/18 |
| 524, OB132_light | R.TEGIFR.L | 722.38 | | 1 | 1.52 | 0.15 | 168.9 | 2 | 7/10 |

| **AN02142 (KapA) -- 60589.8 -- 4.9 -- 16.3%** | | | | | | **80.3** |  | **8 (8-0-0-0-0)** |
| --- | --- | --- | --- | --- | --- | --- | --- | --- |
| 347, OB131_light | K.VGEMDKEAGQGDAHVNR.Y | 1812.83 | 3 | 5.64 | 0.65 | 2588.4 | 1 | 33/64 |
| 842, OB130_light | R.EQAVWALGNIAGDSPQCR.D | 1971.93 | 2 | 4.49 | 0.67 | 1962.6 | 1 | 21/34 |
| 832, OB131_light | R.YALFIEEAGGMEK.I | 1457.70 | 2 | 3.81 | 0.58 | 1811.4 | 1 | 20/24 |
| 464, OB130_light | R.VIETGVVSR.F | 959.55 | 2 | 2.90 | 0.39 | 1045.2 | 1 | 15/16 |
| 855, OB131_light | K.GVFSDQIEAQIQATTK.F | 1735.89 | 2 | 3.49 | 0.61 | 974.3 | 1 | 18/30 |
| 296, OB130_light | K.VGEM*DKEAGQGDAHVNR.Y | 1828.83 | 3 | 3.20 | 0.51 | 1224.7 | 1 | 29/64 |
| 503, OB130_light | R.NQFRPDELR.R | 1174.60 | 2 | 2.57 | 0.08 | 588.1 | 2 | 11/16 |
| 419, OB130_light | R.EEQQVEIR.K | 1030.52 | 2 | 2.46 | 0.22 | 473.4 | 1 | 11/14 |
