# Supplementary material for: LaeA Control of Velvet Family Regulatory Proteins for Light-Dependent Development and Fungal Cell-Type Specificity
Source: PLoS Genet. 2010 Dec 2;6(12):e1001226. doi: 10.1371/journal.pgen.1001226 (PMC2996326; doi:10.1371/journal.pgen.1001226)
Supplement: Table S7 — SEQUEST Multiple Consensus Report of VelB::cTAP tag identifications in laeAΔ after nano-LC-ESI-MS2. (0.19 MB DOC) [file pgen.1001226.s013.doc]

**Table S7.** **SEQUEST Multiple Consensus Report of VelB::cTAP tag identifications in *laeA*∆ after nano-LC-ESI-MS2.**

A) Dark cultures

| **Reference (database entry) -- Average Mass -- pI -- Coverage (amino acids)** | | | | | | **Score** |  | **Peptides** |
| --- | --- | --- | --- | --- | --- | --- | --- | --- |
| **File, Scan(s)** | **Sequence** | **MH+** | **Charge** | **XCorr** | **Delta Cn** | **Sp** | **RSp** | **Ions** |
| **AN1959 (VosA) -- 48980.2 -- 8.8 -- 34.4%** | | | | | | **118.2** |  | **12 (11-1-0-0-0)** |
| 784, OB2_dark | R.TAMQIPGSSYPAPPYQPTSR.D | 2149.03 | 2 | 4.56 | 0.57 | 833.0 | 1 | 21/38 |
| 635, OB3_dark | R.TAM*QIPGSSYPAPPYQPTSR.D | 2165.03 | 2 | 3.68 | 0.62 | 936.4 | 1 | 22/38 |
| 1128, OB3_dark | K.DVDNTDGGFFVWGDLSIK.V | 1984.92 | 2 | 4.70 | 0.69 | 1159.7 | 1 | 20/34 |
| 608, OB2_dark | R.TAPRPEEYPQAAIPR.S | 1695.88 | 3 | 3.73 | 0.36 | 1343.4 | 1 | 26/56 |
| 814, OB3_dark | R.PSTSDDFELIVR.Q | 1378.68 | 2 | 4.09 | 0.54 | 2071.1 | 1 | 19/22 |
| 604, OB3_dark | R.KPVDPPPIVQIR.V | 1358.81 | 3 | 4.58 | 0.41 | 1719.3 | 1 | 27/44 |
| 818, OB2_dark | R.GYYEQSPQATPILPSQPLGTSEAER.Y | 2719.32 | 2 | 3.68 | 0.59 | 386.4 | 1 | 18/48 |
| 519, OB3_dark | R.FTVSPPK.S | 775.43 | 1 | 1.85 | 0.20 | 255.6 | 1 | 8/12 |
| 459, OB1_dark | R.YGVPPGHTGYDHTGSANGTPR.- | 2140.97 | 3 | 1.89 | 0.26 | 478.4 | 1 | 26/80 |
| 640, OB3_dark | R.DYSYYAPVK.R | 1105.52 | 2 | 2.85 | 0.53 | 651.3 | 1 | 12/16 |
| 684, OB3_dark | R.TDVVFLK.S | 821.47 | 1 | 1.74 | 0.26 | 455.3 | 2 | 9/12 |
| 894, OB3_dark | K.FSLFEMR.K | 929.45 | 1 | 1.24 | 0.28 | 126.5 | 1 | 8/12 |
| **AN0363 (VelB) -- 40015.7 -- 6.1 -- 46.6%** | | | | | | **168.3** |  | **17 (16-1-0-0-0)** |
| 902, OB2_dark | K.SVSDLPQSDIAEVINK.G | 1714.88 | 2 | 4.54 | 0.62 | 1003.5 | 1 | 24/30 |
| 1038, OB3_dark | K.GTAPILASTFSEPFQVFSAK.K | 2098.08 | 3 | 4.59 | 0.56 | 2122.6 | 1 | 35/76 |
| 732, OB2_dark | K.KFPGVIESTPLSK.V | 1402.79 | 2 | 3.14 | 0.55 | 772.7 | 1 | 18/24 |
| 1317, OB2_dark | K.EVDINSLDSSFYVVMADLWNADGTHEVNLVK.H | 3480.66 | 3 | 4.37 | 0.53 | 765.7 | 1 | 32/120 |
| 907, OB2_dark | R.IWSLQVVQQPIR.A | 1466.84 | 2 | 4.33 | 0.39 | 2131.3 | 1 | 18/22 |
| 1168, OB2_dark | K.EVDINSLDSSFYVVM*ADLWNADGTHEVNLVK.H | 3496.66 | 3 | 5.39 | 0.62 | 901.5 | 1 | 32/120 |
| 823, OB1_dark | R.NLIGCLSASAYR.L | 1324.66 | 2 | 3.89 | 0.63 | 1450.8 | 1 | 19/22 |
| 1247, OB2_dark | K.IGVWFVLQDLSVR.T | 1531.86 | 2 | 4.14 | 0.39 | 1707.3 | 1 | 18/24 |
| 813, OB2_dark | K.FPGVIESTPLSK.V | 1274.69 | 2 | 3.33 | 0.54 | 1085.8 | 1 | 18/22 |
| 718, OB3_dark | K.FSFVNVGK.S | 897.48 | 2 | 2.27 | 0.39 | 591.0 | 1 | 12/14 |
| 437, OB1_dark | R.MCGFGDKDR.R | 1085.45 | 2 | 2.91 | 0.48 | 1050.8 | 1 | 14/16 |
| 383, OB2_dark | R.AHSGHHPPPLSMDR.I | 1538.72 | 2 | 2.57 | 0.36 | 314.9 | 1 | 13/26 |
| 457, OB3_dark | R.RPITPPPCIR.L | 1206.67 | 2 | 2.25 | 0.49 | 379.5 | 1 | 12/18 |
| 298, OB2_dark | R.AHSGHHPPPLSM*DR.I | 1554.72 | 2 | 2.29 | 0.45 | 325.2 | 1 | 13/26 |
| 443, OB3_dark | K.VFANQGIK.I | 876.49 | 1 | 1.64 | 0.12 | 526.3 | 1 | 11/14 |
| 537, OB3_dark | R.TEGIFR.L | 722.38 | 1 | 1.38 | 0.19 | 208.9 | 5 | 7/10 |
| 475, OB2_dark | R.M*CGFGDKDR.R | 1101.44 | 2 | 2.10 | 0.23 | 638.6 | 1 | 12/16 |

| **Reference (database entry) -- Average Mass -- pI -- Coverage (amino acids)** | | | | | | | **Score** |  | **Peptides** |
| --- | --- | --- | --- | --- | --- | --- | --- | --- | --- |
| **File, Scan(s)** | **Sequence** | **MH+** | **Charge** | | **XCorr** | **Delta Cn** | **Sp** | **RSp** | **Ions** |
| **AN1052 (VeA) -- 59282.3 -- 9.5 -- 44.9%** | | | | | | | **250.3** |  | **25 (25-0-0-0-0)** |
| 862, OB1_dark | K.LMTNQGSPVLTGVPVAGVAYLDKPNR.A | 2697.43 | | 3 | 4.97 | 0.64 | 882.2 | 1 | 35/100 |
| 727, OB1_dark | R.NQSISEYEPSMGYPGSQTR.L | 2130.93 | | 2 | 4.96 | 0.67 | 1070.0 | 1 | 23/36 |
| 683, OB1_dark | R.PSYGQPSQTTSLPPLR.H | 1728.89 | | 2 | 4.11 | 0.64 | 655.8 | 1 | 19/30 |
| 612, OB1_dark | R.STSISTNM*DPYSYPSR.R | 1821.80 | | 2 | 3.84 | 0.73 | 771.6 | 1 | 15/30 |
| 434, OB3_dark | R.RPDQYAGSDAYANAPERPR.S | 2134.00 | | 3 | 5.53 | 0.57 | 1410.1 | 1 | 32/72 |
| 712, OB1_dark | R.STSISTNMDPYSYPSR.R | 1805.80 | | 2 | 4.44 | 0.72 | 1165.9 | 1 | 19/30 |
| 582, OB1_dark | R.RPSAVEYGQPIAQPYQR.P | 1960.00 | | 2 | 4.42 | 0.65 | 760.7 | 1 | 19/32 |
| 1007, OB2_dark | R.LEVISNPFIVYSAK.K | 1579.87 | | 2 | 4.20 | 0.57 | 1138.7 | 1 | 19/26 |
| 1017, OB3_dark | R.AGYFIFPDLSVR.N | 1384.72 | | 2 | 3.35 | 0.43 | 960.4 | 1 | 18/22 |
| 834, OB1_dark | K.LM*TNQGSPVLTGVPVAGVAYLDKPNR.A | 2713.43 | | 2 | 3.82 | 0.62 | 483.5 | 1 | 20/50 |
| 428, OB1_dark | K.RTEDYDYDNER.G | 1475.60 | | 2 | 3.60 | 0.52 | 1332.0 | 1 | 17/20 |
| 748, OB1_dark | R.LWETNSMLSK.R | 1208.59 | | 2 | 3.10 | 0.36 | 660.3 | 1 | 15/18 |
| 652, OB1_dark | K.KFPGLTTSTPISR.M | 1404.78 | | 2 | 2.66 | 0.54 | 775.6 | 1 | 17/24 |
| 758, OB1_dark | R.SSLLDGPDQMAYK.R | 1424.67 | | 2 | 3.89 | 0.35 | 1409.7 | 1 | 19/24 |
| 642, OB1_dark | R.NQSISEYEPSM*GYPGSQTR.L | 2146.93 | | 2 | 4.00 | 0.65 | 619.9 | 1 | 19/36 |
| 492, OB1_dark | R.TEDYDYDNER.G | 1319.50 | | 2 | 3.38 | 0.58 | 1113.6 | 1 | 16/18 |
| 885, OB1_dark | R.FSFHLFEQIK.D | 1295.67 | | 2 | 2.98 | 0.39 | 1275.0 | 1 | 15/18 |
| 808, OB1_dark | R.FSFHLFEQIKDPK.D | 1635.85 | | 2 | 3.78 | 0.44 | 626.8 | 1 | 17/24 |
| 624, OB2_dark | K.DATEGTQPMPSPVPGK.L | 1611.7 | | 2 | 3.63 | 0.63 | 1139.1 | 1 | 22/30 |
| 947, OB2_dark | K.LSSPQEFLEFR.L | 1352.68 | | 2 | 2.74 | 0.47 | 543.3 | 1 | 14/20 |
| 653, OB1_dark | R.LSAERPSYGQPSQTTSLPPLR.H | 2285.18 | | 3 | 4.60 | 0.56 | 1047.2 | 1 | 28/80 |
| 714, OB3_dark | K.FPGLTTSTPISR.M | 1276.68 | | 2 | 3.21 | 0.43 | 887.7 | 1 | 16/22 |
| 488, OB1_dark | K.DATEGTQPM*PSPVPGK.L | 1627.76 | | 2 | 3.77 | 0.56 | 665.0 | 1 | 18/30 |
| 669, OB1_dark | R.SSLLDGPDQM*AYK.R | 1440.67 | | 2 | 3.11 | 0.38 | 783.9 | 1 | 17/24 |
| 417, OB2_dark | R.HSLEPSVNSR.S | 1125.56 | | 2 | 2.54 | 0.48 | 253.0 | 2 | 10/18 |

B) **Light cultures**

| **Reference (database entry) -- Average Mass -- pI -- Coverage (amino acids)** | | | | | | **Score** |  | **Peptides** |
| --- | --- | --- | --- | --- | --- | --- | --- | --- |
| **File, Scan(s)** | **Sequence** | **MH+** | **Charge** | **XCorr** | **Delta Cn** | **Sp** | **RSp** | **Ions** |
| **AN1959 (VosA) -- 48980.2 -- 8.8 -- 33.7%** | | | | | | **128.2** |  | **12 (11-1-0-0-0)** |
| 670, OB5_light | R.TAM*QIPGSSYPAPPYQPTSR.D | 2165.04 | 2 | 4.20 | 0.56 | 665.6 | 1 | 21/38 |
| 754, OB6_light | R.TAMQIPGSSYPAPPYQPTSR.D | 2149.04 | 2 | 3.76 | 0.57 | 930.3 | 1 | 23/38 |
| 727, OB5_light | R.PQYSASTAVLPPLQQSR.N | 1842.97 | 2 | 4.44 | 0.58 | 1029.2 | 1 | 24/32 |
| 579, OB6_light | R.TAPRPEEYPQAAIPR.S | 1695.88 | 3 | 3.41 | 0.53 | 1035.5 | 1 | 24/56 |
| 1154, OB6_light | K.DVDNTDGGFFVWGDLSIK.V | 1984.93 | 2 | 3.98 | 0.64 | 934.4 | 1 | 17/34 |
| 864, OB5_light | K.SFPGMAESTFLSR.S | 1429.68 | 2 | 3.07 | 0.56 | 460.6 | 1 | 19/24 |
| 638, OB6_light | R.KPVDPPPIVQIR.V | 1358.82 | 3 | 4.30 | 0.21 | 2234.3 | 1 | 27/44 |
| 664, OB5_light | R.DYSYYAPVK.R | 1105.52 | 1 | 2.55 | 0.46 | 554.0 | 1 | 11/16 |
| 927, OB6_light | K.FSLFEMR.K | 929.45 | 2 | 2.91 | 0.38 | 1015.4 | 1 | 12/12 |
| 547, OB5_light | R.FTVSPPK.S | 775.43 | 1 | 1.83 | 0.23 | 269.3 | 1 | 8/12 |
| 655, OB5_light | R.SSQQATM*QSLGMVNPPGTPTPDSAR.A | 2574.20 | 2 | 3.77 | 0.30 | 714.5 | 1 | 20/48 |
| 655, OB5_light | R.SSQQATMQSLGM*VNPPGTPTPDSAR.A | 2574.20 | 2 | 2.66 | 0.58 | 347.4 | 2 | 15/48 |
| 715, OB6_light | R.TDVVFLK.S | 821.48 | 1 | 1.84 | 0.34 | 383.7 | 1 | 9/12 |
| **AN0363 (VelB) -- 40015.7 -- 6.1 -- 49.3%** | | | | | | **160.3** |  | **16 (16-0-0-0-0)** |
| 807, OB5_light | K.SVSDLPQSDIAEVINK.G | 1714.89 | 2 | 5.01 | 0.59 | 1123.1 | 1 | 25/30 |
| 1067, OB5_light | K.GTAPILASTFSEPFQVFSAK.K | 2098.09 | 3 | 4.50 | 0.44 | 1419.1 | 1 | 32/76 |
| 1262, OB5_light | K.EVDINSLDSSFYVVMADLWNADGTHEVNLVK.H | 3480.66 | 3 | 4.76 | 0.47 | 965.7 | 1 | 35/120 |
| 800, OB5_light | R.NLIGCLSASAYR.L | 1324.67 | 2 | 3.85 | 0.55 | 1501.9 | 1 | 19/22 |
| 862, OB5_light | R.IWSLQVVQQPIR.A | 1466.85 | 2 | 4.05 | 0.40 | 2072.5 | 1 | 18/22 |
| 1162, OB6_light | K.IGVWFVLQDLSVR.T | 1531.86 | 2 | 4.49 | 0.46 | 2009.4 | 1 | 19/24 |
| 1134, OB5_light | K.EVDINSLDSSFYVVM*ADLWNADGTHEVNLVK.H | 3496.66 | 3 | 3.77 | 0.45 | 1029.2 | 1 | 32/120 |
| 687, OB5_light | K.KFPGVIESTPLSK.V | 1402.79 | 2 | 3.16 | 0.46 | 643.5 | 1 | 18/24 |
| 342, OB5_light | R.AHSGHHPPPLSMDR.I | 1538.73 | 3 | 2.86 | 0.42 | 916.2 | 1 | 23/52 |
| 403, OB5_light | R.MCGFGDKDR.R | 1085.45 | 2 | 2.68 | 0.49 | 783.0 | 1 | 12/16 |
| 759, OB5_light | K.FPGVIESTPLSK.V | 1274.70 | 2 | 3.12 | 0.47 | 1006.1 | 1 | 18/22 |
| 753, OB6_light | K.FSFVNVGK.S | 897.48 | 2 | 2.18 | 0.29 | 669.0 | 1 | 13/14 |
| 520, OB5_light | K.VFANQGIK.I | 876.49 | 1 | 1.89 | 0.20 | 539.0 | 1 | 10/14 |
| 472, OB5_light | R.RPITPPPCIR.L | 1206.68 | 2 | 2.94 | 0.32 | 571.5 | 1 | 14/18 |
| 358, OB5_light | R.LIVKDAQTQK.E | 1143.67 | 2 | 2.16 | 0.25 | 486.4 | 1 | 11/18 |
| 568, OB6_light | R.TEGIFR.L | 722.38 | 1 | 1.67 | 0.14 | 282.3 | 1 | 8/10 |

| **Reference (database entry) -- Average Mass -- pI -- Coverage (amino acids)** | | | | | | | | **Score** |  | **Peptides** |
| --- | --- | --- | --- | --- | --- | --- | --- | --- | --- | --- |
| **File, Scan(s)** | | **Sequence** | **MH+** | **Charge** | | **XCorr** | **Delta Cn** | **Sp** | **RSp** | **Ions** |
| **AN1052 (VeA) -- 59282.3 -- 9.5 -- 46.0%** | | | | | | | | **250.3** |  | **25 (25-0-0-0-0)** |
| 702, OB4_light | R.NQSISEYEPSMGYPGSQTR.L | | 2130.94 | | 2 | 4.28 | 0.69 | 599.6 | 1 | 18/36 |
| 473, OB6_light | R.RPDQYAGSDAYANAPERPR.S | | 2134.01 | | 3 | 4.89 | 0.62 | 1525.4 | 1 | 33/72 |
| 963, OB4_light | R.LEVISNPFIVYSAK.K | | 1579.87 | | 2 | 4.32 | 0.56 | 1338.9 | 1 | 21/26 |
| 677, OB4_light | R.PSYGQPSQTTSLPPLR.H | | 1728.89 | | 2 | 4.38 | 0.64 | 792.5 | 1 | 20/30 |
| 1037, OB5_light | R.AGYFIFPDLSVR.N | | 1384.73 | | 2 | 3.50 | 0.53 | 916.8 | 1 | 17/22 |
| 832, OB6_light | K.LM*TNQGSPVLTGVPVAGVAYLDKPNR.A | | 2713.44 | | 3 | 4.16 | 0.58 | 656.3 | 1 | 30/100 |
| 845, OB5_light | K.LMTNQGSPVLTGVPVAGVAYLDKPNR.A | | 2697.44 | | 2 | 3.67 | 0.45 | 327.3 | 1 | 17/50 |
| 697, OB5_light | R.STSISTNMDPYSYPSR.R | | 1805.80 | | 2 | 4.31 | 0.66 | 1078.8 | 1 | 17/30 |
| 637, OB5_light | K.KFPGLTTSTPISR.M | | 1404.78 | | 2 | 3.14 | 0.53 | 805.1 | 1 | 18/24 |
| 652, OB4_light | R.SSLLDGPDQM*AYK.R | | 1440.67 | | 2 | 3.52 | 0.42 | 1127.9 | 1 | 19/24 |
| 567, OB4_light | R.RPSAVEYGQPIAQPYQR.P | | 1960.00 | | 2 | 4.75 | 0.64 | 1023.1 | 1 | 24/32 |
| 897, OB4_light | K.LSSPQEFLEFR.L | | 1352.68 | | 2 | 3.04 | 0.49 | 795.5 | 1 | 15/20 |
| 632, OB4_light | R.NQSISEYEPSM*GYPGSQTR.L | | 2146.94 | | 3 | 4.76 | 0.67 | 1293.1 | 1 | 31/72 |
| 732, OB4_light | R.LWETNSMLSK.R | | 1208.60 | | 2 | 3.22 | 0.44 | 615.7 | 1 | 15/18 |
| 479, OB6_light | R.TEDYDYDNER.G | | 1319.50 | | 2 | 2.90 | 0.51 | 743.0 | 1 | 14/18 |
| 597, OB4_light | R.STSISTNM*DPYSYPSR.R | | 1821.80 | | 2 | 3.33 | 0.56 | 969.8 | 1 | 16/30 |
| 407, OB4_light | K.RTEDYDYDNER.G | | 1475.60 | | 2 | 3.20 | 0.42 | 1057.2 | 1 | 15/20 |
| 475, OB5_light | K.DATEGTQPM*PSPVPGK.L | | 1627.77 | | 2 | 3.63 | 0.62 | 888.5 | 1 | 20/30 |
| 593, OB5_light | K.DATEGTQPMPSPVPGK.L | | 1611.77 | | 2 | 3.51 | 0.56 | 709.9 | 1 | 18/30 |
| 619, OB4_light | R.LWETNSM*LSK.R | | 1224.60 | | 2 | 2.73 | 0.44 | 657.1 | 1 | 15/18 |
| 640, OB4_light | R.LSAERPSYGQPSQTTSLPPLR.H | | 2285.19 | | 2 | 3.77 | 0.65 | 615.3 | 1 | 18/40 |
| 742, OB4_light | K.FPGLTTSTPISR.M | | 1276.69 | | 2 | 3.08 | 0.58 | 801.2 | 1 | 16/22 |
| 352, OB4_light | R.MVSKPATMR.- | | 1020.53 | | 2 | 2.31 | 0.26 | 434.3 | 1 | 12/16 |
| 377, OB4_light | R.HSLEPSVNSR.S | | 1125.56 | | 2 | 2.31 | 0.39 | 304.9 | 1 | 10/18 |
| 890, OB4_light | R.FSFHLFEQIK.D | | 1295.68 | | 2 | 2.76 | 0.34 | 981.3 | 1 | 15/18 |
